# An early Sox2-dependent gene expression programme required for hippocampal dentate gyrus development

**DOI:** 10.1098/rsob.200339

**Published:** 2021-02-24

**Authors:** Sara Mercurio, Chiara Alberti, Linda Serra, Simone Meneghini, Pietro Berico, Jessica Bertolini, Andrea Becchetti, Silvia K. Nicolis

**Affiliations:** Department of Biotechnology and Biosciences, University of Milano-Bicocca, Piazza della Scienza 2, 20126 Milano, Italy

**Keywords:** transcription factors, Sox, Sox2, mouse genetic models, gene regulation

## Abstract

The hippocampus is a brain area central for cognition. Mutations in the human SOX2 transcription factor cause neurodevelopmental defects, leading to intellectual disability and seizures, together with hippocampal dysplasia. We generated an allelic series of Sox2 conditional mutations in mouse, deleting Sox2 at different developmental stages. Late Sox2 deletion (from E11.5, via Nestin-Cre) affects only postnatal hippocampal development; earlier deletion (from E10.5, Emx1-Cre) significantly reduces the dentate gyrus (DG), and the earliest deletion (from E9.5, FoxG1-Cre) causes drastic abnormalities, with almost complete absence of the DG. We identify a set of functionally interconnected genes (Gli3, Wnt3a, Cxcr4, p73 and Tbr2), known to play essential roles in hippocampal embryogenesis, which are downregulated in early Sox2 mutants, and (Gli3 and Cxcr4) directly controlled by SOX2; their downregulation provides plausible molecular mechanisms contributing to the defect. Electrophysiological studies of the Emx1-Cre mouse model reveal altered excitatory transmission in CA1 and CA3 regions.

## Graphical abstract

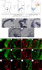

## 1. Introduction

The hippocampus is a brain region important for cognition, playing essential roles in learning and in spatial and episodic memory formation. Hippocampus defects (of genetic origin, or acquired) can lead to intellectual disability (ID), deficits of memory formation and epilepsy [1].

Within the hippocampus, the dentate gyrus (DG) represents the primary input site for excitatory neuronal projections; the major type of DG neurons (granule neurons) are generated by neural stem cells (NSC) that are defined early in development, and continue neurogenesis during embryogenesis and also in postnatal stages, in mice as well as in humans [2,3].

Patients carrying heterozygous loss-of-function mutations in the gene encoding the SOX2 transcription factor show a characteristic spectrum of central nervous system (CNS) defects, including hippocampal defects (involving the DG), ID and epilepsy [4–7]. Understanding the developmental events and the genetic programme controlled by SOX2 during hippocampal embryogenesis, therefore, provides a key to understand how their perturbation can lead to hippocampal disease (in SOX2-mutant patients and, more in general, in hippocampal defects of genetic origin).

In mouse, Sox2-dependent hippocampal disease has been previously modelled by conditional mutagenesis [8]. The mouse phenotype in heterozygous individuals is much milder than that in human heterozygous patients, whereas in homozygous conditional knock-out mutant mice, hippocampal and eye defects can be observed, as in heterozygous humans; this points to a differential sensitivity to SOX2 dosage in mice versus humans, whose molecular basis is still not understood. Sox2 pan-neural deletion at mid-embryogenesis, via a Nestin-Cre transgene, led to a relatively normal hippocampal development up to birth; at early postnatal stages, however, the hippocampus failed to complete its development, and remained hypoplastic, due to a failure of postnatal DG NSC. The study of SOX2 binding to DNA in NSC proved instrumental in the identification of various Sox2 target genes, playing important roles in the development of different brain regions *in vivo*, such as the basal ganglia [9], the cerebellum [10] and the visual thalamus [11,12].

While postnatal hippocampal development was perturbed following Nestin-Cre-mediated Sox2 deletion, embryonic hippocampal development was, quite surprisingly, very little, if at all, affected in these mutants [8]. In principle, this could be due to redundant functions played by other homologous genes of the SoxB family, such as Sox1 and Sox3, coexpressed with Sox2 in the developing neural tube, and reported to function in hippocampal neural stem/progenitor cells [13]; alternatively, we reasoned that Sox2 may play non-redundant, very early functions in hippocampal development, that might not be revealed by Nestin-Cre-mediated deletion.

Here, we generated an allelic series of Sox2 conditional mutations, using Cre transgenes deleting Sox2 at stages earlier than Nestin-Cre: FoxG1-Cre, active from embryonic day (E) 8.5 [14], and Emx1-Cre [15], active from E10.5. We report that early Sox2 deletion leads to drastic defects of hippocampal development, the earlier the deletion, the stronger the phenotype: in Emx1-Cre mutants, hippocampal development is perturbed, but still present, but in FoxG1-Cre mutants, hippocampal development is severely impaired, and the DG essentially fails to develop. We propose that Sox2 sets in motion a very early gene expression programme in the hippocampal primordium, required for all of its subsequent development. Indeed, we show that early (but not late) Sox2 deletion reduces the expression of several genes (some of which SOX2-bound), individually characterized by previous studies as master regulators of hippocampal development (and human neurodevelopmental disease), including Gli3, Wnt3a, Cxcr4, Tbr2 and p73, some of which are known to cross-regulate each other.

## 2. Results

### 2.1. Sox2 is expressed in the primordium of the developing hippocampus and in the adjacent cortical hem

**Figure 1.**
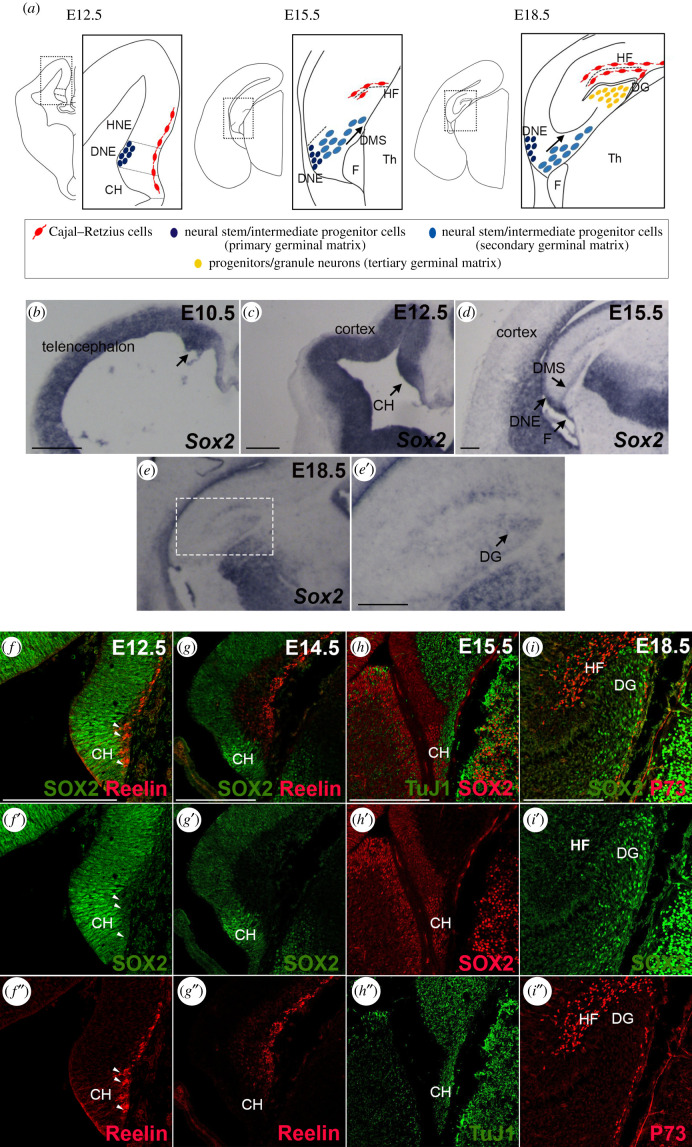
Sox2 expression in the dorsal telencephalon. (*a*) Schematic of the development of the hippocampus in the dorsal telencephalon. (*b*–*e*) ISH for *Sox2* on coronal section of mouse brains at E10.5 (*b*) E12.5 (*c*), E15.5 (*d*) and E18.5 (*e*). Arrows indicate Sox2 expression in the developing hippocampus in particular in the dorsal telencephalon in (*b*), in the CH in (*c*), in the dorsal migratory stream (DMS) in (*d*) and in the DG in (*e*′). (*f*–*i*) IF of Sox2 (*f*–*i*), of markers of CRC, Reelin (*f*,*g*) and P73 (*i*), and of a marker of differentiating neurons TuJ1 (*h*). Representative single optical confocal sections are shown. Scale bars 200 µm. CH, cortical hem; DNE, dentate neuroepithelium; HNE, hippocampal neuroepithelium; DMS, dentate migratory stream; HF, hippocampal fissure; DG, dentate gyrus; F, fimbria; Th, thalamus.

The transcription factor Sox2 is expressed throughout the neural tube from the beginning of its development [8,16–18]. The hippocampus starts to develop around embryonic day (E) 12.5, in the medial wall of the telencephalon, and becomes morphologically recognizable in the following days (figure 1*a*) [2,19]. A region essential for the formation of the hippocampus is the cortical hem (CH), also known as the hippocampal organizer, identified in mice at E12.5; signalling from the CH is able to organize the surrounding tissue into a hippocampus [20,21]. The dentate neural epithelium (DNE), adjacent to the CH (figure 1*a*), contains NSC, that will generate granule neurons in the hippocampus DG throughout development and, subsequently, in postnatal life [2]. On the outer side of the neuroepithelium, towards the pia, a population of neurons, called Cajal–Retzius cells (CRC) (figure 1*a*) develops, that will have a key role in the morphogenesis of the hippocampus. NSC and intermediate neural progenitors (INP) will migrate from the DNE, along the dentate migratory stream (DMS), towards the forming hippocampal fissure (HF), a folding of the meninges that will be invaded by CRC (figure 1*a*).

We examined Sox2 expression by *in situ* hybridization (ISH) and immunofluorescence (IF), in the medial telencephalon, from which the hippocampus develops, between E12.5 and E18.5 (figure 1*b–i*). At E10.5, *Sox2* is expressed in the whole telencephalon including the dorsomedial region that will give rise to the hippocampus (figure 1*b*). At E12.5, *Sox2* is expressed throughout the neuroepithelium in the medial telencephalic wall and it is enriched in the CH region (figure 1*c*); at E15.5, expression persists in the neuroepithelium, and is detected in the DMS and in the fimbria (a CH derivative) (figure 1*d*). Just before birth, at E18.5, *Sox2* expression is detected in the developing DG (figure 1*e,e*′).

We then performed co-immunohistochemistry experiments with antibodies against SOX2, and markers of more differentiated cell types: CR cells markers Reelin and P73 (figure 1*f*,*g*,*i*) and the pan-neuronal marker TuJ1 (figure 1*h*). While SOX2 was detected in all cells within the neuroepithelium, as expected, we detected no or very little (figure 1*f* arrowheads) overlap with TuJ1, Reelin or p73 (figure 1*f*–*i*). Moreover, to test if Sox2 is expressed in the progenitors of CRC, we turned on EYFP in Sox2-expressing cells of the early telencephalon before CRC differentiation started, at E9.5 (via a Sox2-CreERT2 transgene and a lox-stop-lox reporter of Cre activity, electronic supplementary material, figure S1), and found that these cells differentiated into Reelin-expressing CRC in the hippocampal fissure and the cortex (electronic supplementary material, figure S1).

Thus, Sox2 expression in the developing hippocampus and CH is present mainly in undifferentiated neuroepithelial cells (including CRC precursors), and becomes extinguished in differentiation.

### 2.2. Sox2 early ablation (FoxG1-Cre) prevents the development of the hippocampal dentate gyrus, and severely compromises hippocampal embryogenesis

Sox2 is required for postnatal development of the hippocampus, in particular to maintain NSC in the DG [8]; however, whether Sox2 has a role in hippocampus embryogenesis was not known. To address this question, we generated three different conditional knock-outs, to ablate Sox2 at different time points of telencephalon development. Specifically, we crossed a Sox2 floxed allele [8] with the following Cre lines: FoxG1-Cre, deleting between E8.5 and E9.5 [9,14], Emx1-Cre, deleting from E10.5 (though not yet at E9.5) [15,22] and, as a control, Nestin-Cre, deleting after E11.5 [8,23]. The resulting conditional knock-outs (Sox2^flox/flox^;FoxG1-Cre, Sox2^flox/flox^;Emx1-Cre, Sox2^flox/ßgeo^; Nestin-Cre) will be called FoxG1-Cre cKO, Emx1-Cre cKO and Nestin-Cre cKO, respectively, from now onwards. As expected, complete Sox2 deletion is observed by E9.5 in FoxG1-Cre cKO (in the whole telencephalon), and at E10.5 in Emx1-Cre cKO (in the dorsal telencephalon); in the Nestin-Cre cKO, deletion occurs after E11.5 ([8,9]; electronic supplementary material, figure S2).

**Figure 2.**
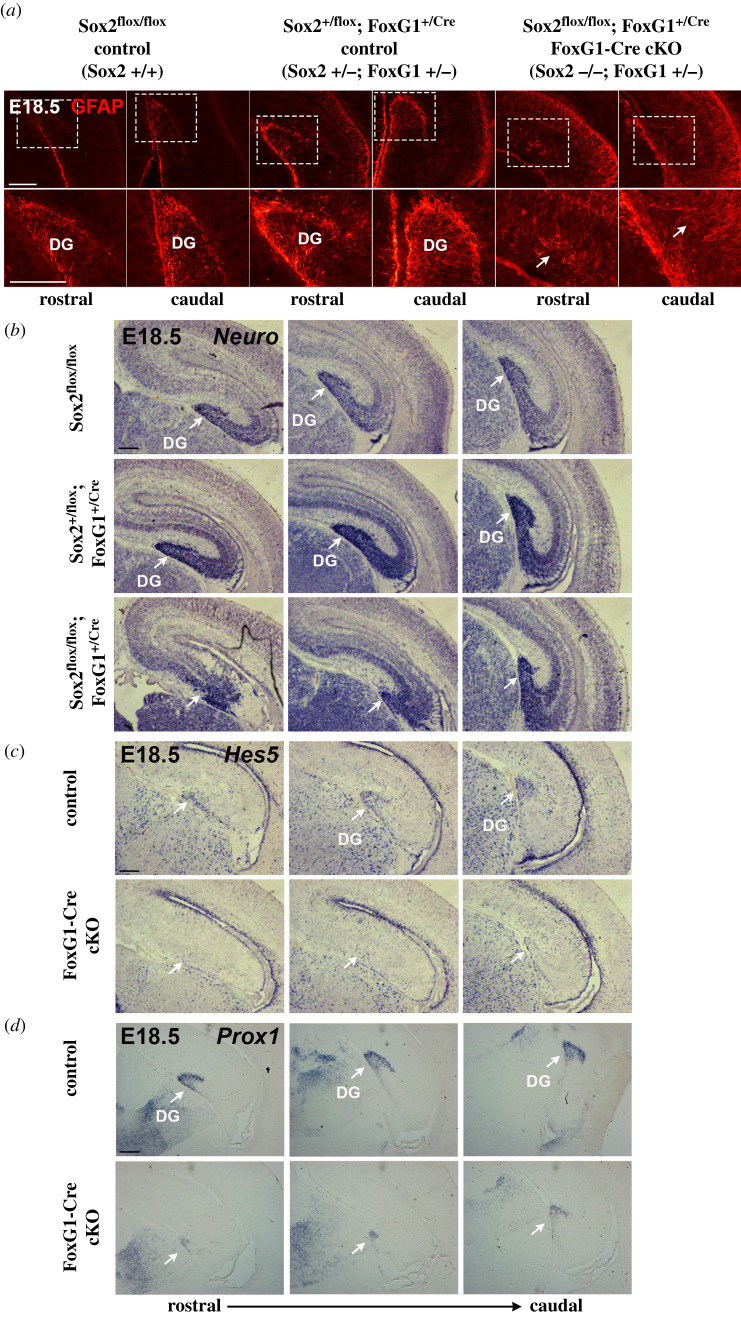
Hippocampal DG development is impaired in FoxG1-Cre cKO Sox2 mutants. (*a*) GFAP IF at E18.5 on coronal sections of control and FoxG1-Cre cKO hippocampi (controls *n* = 7 (Sox2 +/+ *n* = 4, Sox2 +/−; FoxG1 +/− *n* = 3); mutants *n* = 4). (*b*–*d*) ISH at E18.5 for *NeuroD* (*b*) (controls *n* = 4 (Sox2 +/+ *n* = 2, Sox2 +/−; FoxG1 +/− *n* = 2); mutants *n* = 3), *Hes5* (*c*) (controls *n* = 2 (Sox2 +/−; FoxG1 +/− *n* = 2); mutants *n* = 2) and *Prox1* (*d*) (controls *n* = 2; mutants *n* = 2) on coronal sections of control and FoxG1-Cre cKO hippocampi. Arrows indicate the underdeveloped DG in cKO. Scale bars, 200 µm.

**Figure 3.**
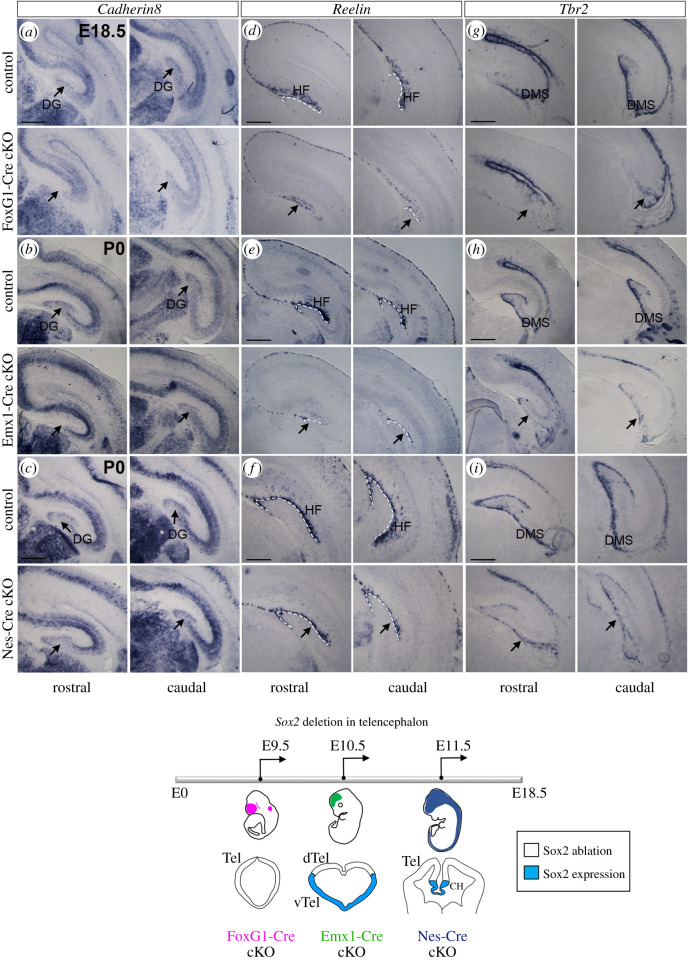
Hippocampus development is affected by Sox2 loss, the earlier Sox2 is ablated the stronger the phenotype observed. ISH for *Cadherin8* (*a*–*c*), *Reelin* (*d*–*f*) and *Tbr2* (*g*–*i*) on coronal sections of control and Sox2 FoxG1-Cre cKO brains at E18.5 (*a*,*d*,*g*), control and Emx1-Cre cKO brains at P0 (*b*,*e*,*h*) and control and Nes-Cre cKO brains at P0 (*c*,*f*,*i*). At least three controls and three mutants were analysed for each probe. A schematic of the timing of Sox2 ablation with the different Cre lines is at the bottom. Scale bars, 200 µm. DG, dentate gyrus; HF, hippocampal fissure; DMS, dentate migratory stream; Tel, telencephalon; dTel, dorsal telencephalon; vTel, ventral telencephalon; CH, cortical hem.

We initially explored hippocampus development in the different mutants at the end of gestation (E18.5; P0) (figures 2 and 3).

We bred Sox2^flox/flox^ to Sox2 ^flox/+^ ;Cre mice, obtaining four different genotypes, each in a 25% proportion: (i) Sox2^flox/flox^; Cre; (ii) Sox2^flox/+^; Cre; (iii) Sox2^flox/flox^; (iv) Sox2^flox/+^. In Sox2^flox/flox^ ;Cre embryos, Sox2 is deleted in homozygosis in the forebrain (mutant genotype); all other genotypes are either wild-type, or carry heterozygous Sox2 deletion.

We first looked at hippocampal development in FoxG1-Cre Sox2 cKO mutants.

We note that the FoxG1-Cre deleter carries the Cre transgene ‘knocked-in’ into the FoxG1 gene, creating a FoxG1 null allele [14]; therefore, Sox2 heterozygous and homozygous mutants both carry, at the same time, a heterozygous FoxG1 mutation (figure 2), which might, in principle, contribute to defective hippocampal development. Previous work showed that FoxG1 heterozygous loss leads, during the course of postnatal life, to some reduction of DG size; however, at the end of embryonic development (E18.5), the authors reported, at most, ‘subtle and inconsistent defects, seen in 50% of the mice’, in hippocampal embryonic development [24], a finding consistent with the previously reported observation that FoxG1 heterozygous mutants are ‘fertile and indistinguishable from wild-type littermates' [25]. In agreement with these observations, we did not observe any significant differences between wild-type embryos and those carrying heterozygous Sox2 deletion, together with FoxG1 heterozygous loss (figure 2*a*,*b*; electronic supplementary material, figure S3 and not shown). Therefore, hereafter, we will call all these mice ‘controls’. The absence of major defects in Sox2+/−; FoxG1+/− mice is to be contrasted with the drastic defects present in Sox2−/−;FoxG1+/− (FoxG1-Cre Sox2 cKO) mutants (see below, figure 2).

We investigated hippocampal development in FoxG1-Cre Sox2 mutants, analysing the development of specific hippocampal cell types, by IF and ISH for cell-type-specific markers at the end of gestation (E18.5). Key for the morphogenesis of the hippocampus is the radial glia (RG) scaffold, marked by GFAP expression, known to be required for the DMS to reach its final destination in the forming DG [26]. By IF for GFAP, at E18.5, we observe a well-defined RG scaffold marking the DG region in both wild-type (Sox2+/+) and heterozygous (Sox2+/−;FoxG1+/−) controls; however, the RG scaffold in the FoxG1-Cre cKO mutant (Sox2−/−; FoxG1+/−) is completely disorganized (figure 2*a*, compare right panels, showing FoxG-Cre cKO mutant, with left panels, showing controls); no morphologically identifiable DG is present in FoxG1-Cre mutants, and the few RG found have random organization (figure 2*a*, arrows). At this same stage, different neuronal populations are normally found in the hippocampus: granule neurons in the DG, and pyramidal neurons forming the CA1, CA2 and CA3 regions. We performed ISH for NeuroD1, a marker of differentiated neurons; whereas in both controls, NeuroD expression marks a well-developed DG at E18.5, in FoxG1-Cre cKO mutants, while NeuroD1-positive cells in the CA regions are present, NeuroD1-positive cells in the DG, abundant in controls, are almost absent (figure 2*b*).

In the DG, at this stage, neural stem/progenitor cells, marked by the expression of the Hes5 gene [27], are normally present (figure 2*c*, controls); in FoxG1-Cre cKO, however, very few Hes5-positive cells are found (figure 2*c*). Finally, ISH analysis of the expression of DG-specific markers Prox1 (marking dentate granule neurons, and important to specify DG over CA3 cell identity [28] and Ctip2 [29,30]) confirms the absence of a large proportion of cells of the DG (figure 2*d*; electronic supplementary material, figure S4).

In conclusion, early Sox2 loss in the telencephalon (FoxG1-Cre cKO) appears to lead to later reduction (by E18.5) of both differentiated neurons and proliferating neural progenitors; in addition, the radial glia scaffold is completely disorganized.

We further performed ISH with probes identifying hippocampal structures and cell types (figure 3), analysing in parallel FoxG1-Cre, Emx1-Cre and Nestin-Cre Sox2 cKO mutants.

ISH for a general marker of the developing hippocampus, Cadherin 8 [31], shows that, at the end of gestation (E18.5, P0), the DG appears little, if at all, affected in the Nestin-Cre cKO (figure 3*c*), as expected [8]. However, in the Emx1-Cre cKO, the DG is greatly reduced, in particular anteriorly (figure 3*b*); remarkably, in the FoxG1-Cre cKO, the DG appears to be almost absent (figure 3*a*).

At the end of gestation, CRC, expressing Reelin [32] and INP, expressing Tbr2 [19], have a characteristic organization in the hippocampus: CRC are localized around the HF, while INP have migrated from the DNE, by the ventricle, along the DMS, have reached the HF and are found below the CRC layer (figure 1*a*). In the FoxG1-Cre cKO, Reelin expression (marking CRC) is greatly reduced, and an HF is not observed (figure 3*d*); in Emx1-Cre cKO, Reelin is reduced, but the HF is visible (figure 3*e*), and in Nestin-Cre cKO, Reelin appears slightly reduced, but with a normal-looking distribution around the HF (figure 3*f*). Similarly, Tbr2 expression is greatly reduced in FoxG1-Cre cKO; an initial DMS is visible, but no DG is observed (figure 3*g*). Instead, in Emx1-Cre cKO, Tbr2-positive INP have reached the HF, but their abundance is greatly reduced (figure 3*h*). On the other hand, in Nestin-Cre cKO, Tbr2-positive INP appear to have completed their migration, and their abundance seems only slightly, if at all, reduced (figure 3*i*).

To summarize, Sox2 ablation by E9.5 in the telencephalon in FoxG1-Cre cKO results, by the end of gestation, in lack of DG formation, accompanied by a missing HF. Ablation just a day later, in Emx1-Cre cKO, has much less dramatic effects: a hippocampal fissure forms, though CRC and INP are reduced and the DG is much smaller compared to controls. Nestin-Cre cKO appear much less, if at all, affected, as previously published [8]. None of the defects described above was seen in control mice.

### 2.3. The formation of the hippocampal fissure and the dentate migration require Sox2 expression from early developmental stages

After having identified the hippocampal defects present, in our mutants, at the end of gestation, we examined earlier developmental stages, to define the developmental history of the defects. We focused in particular on the FoxG1-Cre mutant, showing the most pronounced abnormalities (see figures 2 and 3).

**Figure 4.**
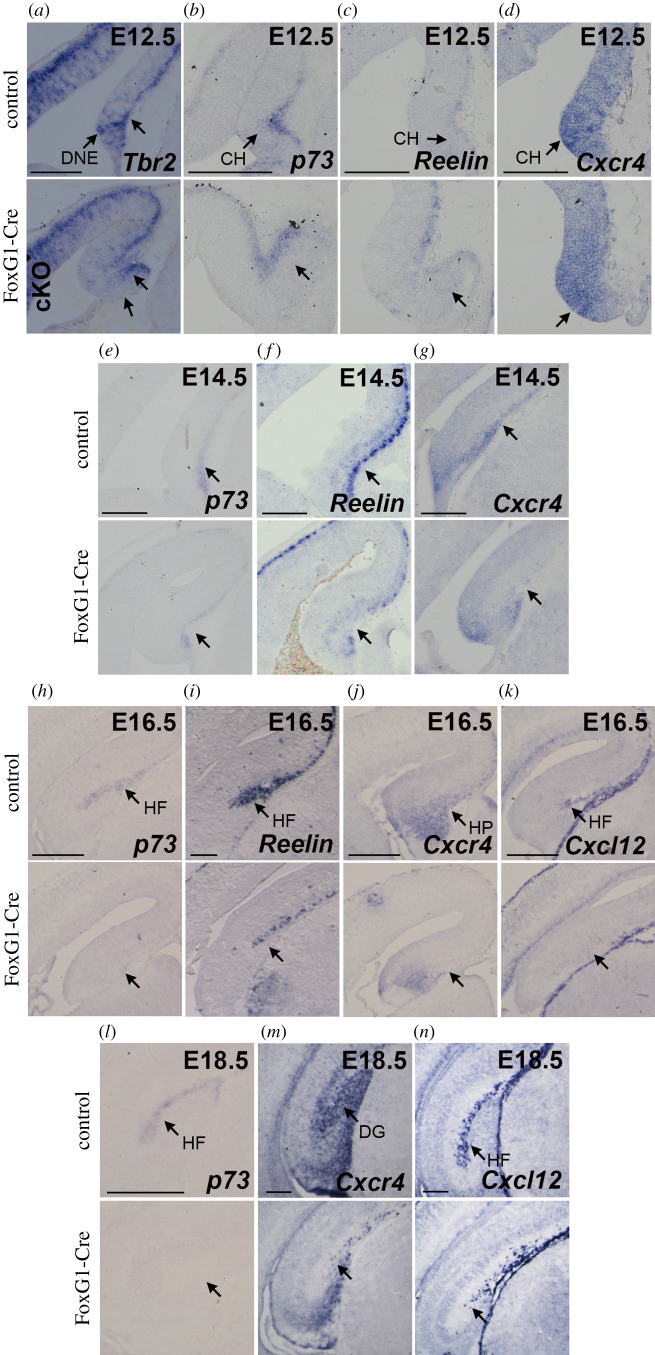
Expression of genes important for the development of the hippocampus is affected by Sox2 loss in FoxG1-Cre cKO. (*a*–*d*) ISH at E12.5 on coronal sections of control and FoxG1-Cre cKO dorsal telencephalons for *Tbr2* (controls *n* = 10, mutants *n* = 10) (*a*)*, P73* (controls *n* = 3, mutants *n* = 3) (*b*), *Reelin* (controls *n* = 7, mutants *n* = 6) (*c*) and *Cxcr4* (controls *n* = 7, mutants *n* = 7) (*d*). (*e*–*g*) ISH at E14.5 on coronal sections of control and FoxG1-Cre cKO brains for *P73* (controls *n* = 3, mutants *n* = 3) (*e*)*, Reelin* (controls *n* = 7, mutants *n* = 5) (*f*) and *Cxcr4* (controls *n* = 3, mutants *n* = 3) (*g*). (*h*–*k*) ISH at E16.5 on coronal sections of control and FoxG1-Cre cKO hippocampi for *P73* (controls *n* = 2, mutants *n* = 2) (*h*), *Reelin* (controls *n* = 6, mutants *n* = 5) (*i*), *Cxcr4* (controls *n* = 5, mutants *n* = 4) (*j*) and *Cxcl12* (controls *n* = 4, mutants *n* = 3) (*k*). (*l*–*n*)) ISH at E18.5 on coronal sections of control and FoxG1-Cre cKO hippocampi for *P73* (controls *n* = 3, mutants *n* = 3) (*l*)*, Cxcr4* (controls *n* = 5, mutants *n* = 4) (*m*) and *Cxcl12* (controls *n* = 5, mutants *n* = 4) (*n*). Arrows indicate the downregulation of expression in the mutant CH, dentate neuroepithelium (DNE), hippocampal primordium (HP), DG and hippocampal fissure (HF). Scale bars, 200 µm.

A defect in the distribution of CRC (marked by Reelin) and INP (marked by Tbr2) is apparent, at the end of gestation, in Sox2 FoxG1-Cre and Emx1-Cre cKO (figure 3*d*,*e*,*g*,*h*). What happens in the first steps of the development of the hippocampus to CRC and INP in these mutants? We addressed this question by ISH with markers for these cell types at early developmental stages, in FoxG1-Cre (Early) cKO embryos (figure 4). We also examined the expression of Cxcr4, a chemokine receptor expressed in INP and neuroblasts in the DMS and in CRC, and its ligand Cxcl12, expressed by the meninges, and required for the migration of INP and CRC [19,26,33,34]; we also examined P73, a P53 homologue, marking CRC and important for hippocampal fissure and DG formation [35,36].

At E12.5, Tbr2 is expressed, in controls, by INP in the DNE and in CR cells towards the pia (figure 4*a*); in the FoxG1-Cre cKO mutant, whereas Tbr2 expression in CRC (towards the pia, arrow) appears present, expression in the DNE is not detected (figure 4*a*). This might reflect a loss of Tbr2-expressing INP; however, we do not observe changes in the number of proliferating cells in this region at E12.5 by EdU labelling (electronic supplementary material, figure S5), suggesting that at least some INP remain, but express less Tbr2 or are mislocalized. P73, Reelin and Cxcr4 expression appears unaltered in mutants compared to controls at this stage (figure 4*b*–*d*).

At E14.5, P73 and Reelin expression marks, in controls, CRC in the medial telencephalic wall region where hippocampal morphogenesis will soon begin (figure 4*e*,*f*, arrow); in the mutant, a strong reduction of P73 and Reelin expression is observed (figure 4*e*,*f*, arrow). Of note, this reduction is detected specifically in the CH of FoxG1-Cre cKO (figure 4*e*,*f*), even though Sox2 is ablated in the whole telencephalon. At this stage, also Cxcr4 expression in CRC appears reduced in the medial telencephalon of FoxG1-Cre cKO (figure 4*g*).

At E16.5, in controls, strong P73 and Reelin expression marks CRC of the hippocampal fissure (HF), defining the beginning of overt hippocampal morphogenesis (figure 4*h,i*); in sharp contrast, this expression is not seen or greatly reduced in the mutant (figure 4*h*,*i*). Cxcl12 is also expressed, in the control, in the developing HF, and its expression is also lost in the mutant (figure 4*k*). Concomitantly, Cxcr4 expression in the hippocampus primordium (HP) is also reduced (figure 4*j*). These data point to a failure to initiate proper HF development in the mutant.

Interestingly, at 16.5, P73, Reelin and Cxcr4 expression is reduced throughout the telencephalon in FoxG1-Cre cKO (figure 4).

At E18.5, P73 marks the HF in controls, but its expression is completely absent in the FoxG1-Cre cKO brain, indicating a complete depletion of P73-positive CH-derived CRC (figure 4*l*). Cxcr4 expression in the DG and Cxcl12 expression in the HF is also greatly reduced in the mutant, confirming a severe abnormality of the mutant hippocampus at the end of gestation (figure 4*m*,*n*).

In conclusion, the defects detected, at the end of gestation, in FoxG1-Cre mutants originate early in development, with a failure, at early stages, to develop an HF and migrating DNE cells in these mutants.

### 2.4. Genes essential for hippocampal development are downregulated following early (FoxG1-Cre cKO), but not late (Emx1-Cre cKO, Nestin-Cre cKO), Sox2 deletion

Having observed that early Sox2 mutants (in particular, FoxG1-Cre cKO) show severely defective hippocampal development, we searched for Sox2-regulated downstream genes, whose deregulation in mutants could explain the observed defects. We compared the expression of several candidate genes in mutants and controls, at E12.5, a stage preceding the observed abnormalities (clearly observed, in mutants, from E14.5, when hippocampal morphogenesis begins). Having observed that the defects in early Sox2 mutants (FoxG1-Cre cKO) are much more severe than those arising in later (Emx1-Cre and Nestin-Cre cKO) mutants, we reasoned that genes downstream to Sox2, that are functionally relevant for these early defects, should show altered expression in early (FoxG1-Cre) mutants, but not, or less, in later mutants (Emx1-Cre; Nestin-Cre).

We thus investigated the expression of genes, representing candidate mediators of Sox2 function, in early and late mutants, by ISH.

Prime candidate genes to mediate defective hippocampal development in early Sox2 mutants include genes encoding signalling molecules, expressed in the CH.

**Figure 5.**
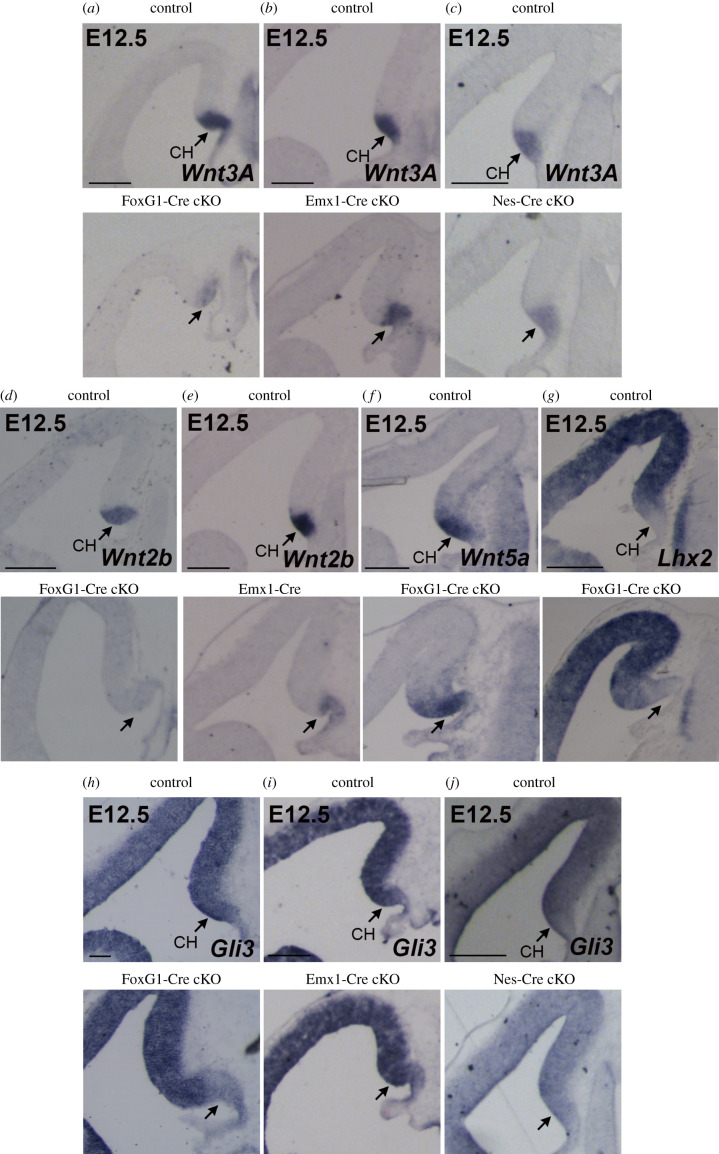
Expression of key molecules for hippocampal development is downregulated in the CH of FoxG1-Cre cKO but mildly or not affected in Emx1-Cre or Nes-Cre cKO. ISH at E12.5 for Wnt3A (*a*–*c*), *Wnt2b* (*d*,*e*), *Wnt5A* (*f*), *Lhx2* (*g*) and Gli3 (*h*–*j*) on control and FoxG1-Cre cKO (*a*,*d*,*f*,*g*,*h*), Emx1-Cre cKO (*b*,*e*,*i*) and Nes-Cre cKO (*c*,*j*) coronal brain sections. Arrows indicate the CH. At least three controls and three mutants were analysed for each probe. Scale bars, 200 µm.

Key signalling molecules secreted by the CH and required for hippocampus formation are components of the Wnt pathway; in fact, Wnt3a knock-out results in a complete loss of the hippocampus [37]. We analysed what happens, at E12.5, to the expression of Wnt3A in the three Sox2 cKO. We found that Wnt3A is severely downregulated specifically in the CH of FoxG1-Cre cKO (figure 5*a*), but only slightly downregulated in Emx1-Cre cKO (figure 5*b*), while it is only very mildly, if at all, reduced at this stage in the Nestin-Cre cKO (figure 5*c*). We analysed the expression of another Wnt family member, Wnt2b in FoxG1-Cre cKO and Emx1-Cre cKO. While Wnt2b was strongly downregulated in the CH of FoxG1-Cre cKO (figure 5*d*), it was only slightly downregulated in the CH of Emx1-Cre cKO compared to controls (figure 5*e*). Wnt5A, another Wnt family member normally expressed in the CH, was instead expressed in the CH of FoxG1-Cre cKO (figure 5*f*), indicating that the CH, as a structure, is present in these mutants, though it fails to express Wnt3a and Wnt2b. Interestingly, expression of the transcription factor Lhx2, a marker of the cortex which is not expressed in the CH, has a normal expression pattern in FoxG1-Cre cKO, including an Lhx2-non-expressing neuroepithelial region, suggesting that a CH is present in these mutants (figure 5*g*).

In conclusion, expression of components of the Wnt pathway known to be involved in the development of the hippocampus is strongly downregulated in the CH of FoxG1-Cre cKO, but not of Emx1-Cre cKO and Nestin-Cre cKO.

Other key genes for hippocampus formation include Gli3, encoding a transcription factor acting as a nuclear effector in the Shh signalling pathway. The knock-out of Gli3 impairs the development of the hippocampus, where DG development is as severely affected as in our Sox2 early (FoxG1-Cre cKO) mutants. Of note, Gli3 acts, in hippocampal development, by regulating expression of components of the Wnt pathway [38]. We found that Gli3 expression is specifically downregulated in the CH (though not in the cortex) of FoxG1-Cre cKO, but not of Emx1-Cre cKO and Nestin-Cre cKO (figure 5*h*–*j*).

**Figure 6.**
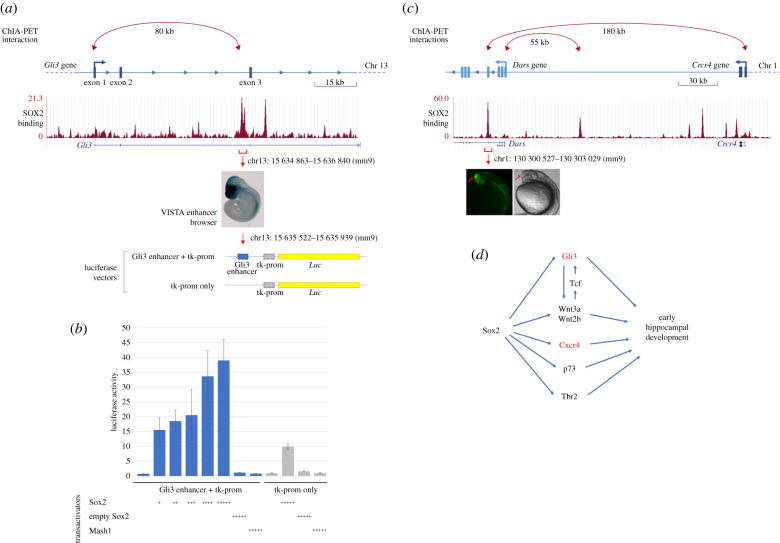
SOX2 acts on distal enhancers and on long-range enhancer–promoter interactions of several genes key to hippocampal development, and activates a Gli3 intronic enhancer in a dose-dependent way. (*a*) Diagram of the Gli3 gene, and SOX2-binding profile across the Gli3 locus in NSC (ChIPseq data from [39]). A Sox2-dependent 80 kb long-range interaction connects the Gli3 promoter with a SOX2-bound region, in the second intron (ChIA-PET data from [39]). This region acts as a brain-specific enhancer in E10.5 mouse embryo (image from https://enhancer.lbl.gov/); it was cloned into the depicted luciferase vector, upstream to a minimal tk promoter, to address its responsivity to Sox2. (*b*) Enhancer activation assay in Neuro2a cells transfected with the constructs in (*a*): Gli3 enhancer + tk promoter (blue histograms), or tk promoter only (grey histograms). Cotransfection of these constructs with increasing amounts of a Sox2-expressing vector (Sox2, *X*-axis), but not of a control ‘empty’ vector (empty Sox2), or a Mash1-expressing vector (Mash1), resulted in dose-dependent increase of luciferase activity (*Y*-axis) driven by the Gli3 enhancer + tk-prom vector, but not the tk-prom only vector. The molar ratios, compared with the luciferase vector (set at 1) were: +, 1 : 0.050; ++, 1 : 0.075; +++, 1 : 0.125; ++++, 1 : 0.25; +++++, 1 : 0.5. Results are represented as fold-change increase in activity compared with the tk-prom only vector, which is set at 1. Values are the mean of two (for Sox2+ and Sox2++) or three (other samples) independent experiments carried out in triplicate. Error bars represent s.d. (*c*) Diagram of the Cxcr4 gene, reporting SOX2 binding and Sox2-dependent long-range interactions in NSC (as in (*a*) for Gli3; data from [39]). Note that the Cxcr4 promoter is connected to a SOX2-bound region within the intron of a different gene, Dars; this region acts as a brain-specific enhancer in transgenic zebrafish embryos (picture from [39]). (*d*) A model depicting the activation, by Sox2, of different genes key to hippocampal development (present paper), some of which cross-regulate each other; in red, direct SOX2 targets; in bold, early expressed hippocampal regulators, downregulated already at early stages in Sox2 mutants (see Discussion).

Recent work from our laboratory identified SOX2-binding sites in an intron of the Gli3 gene in NSC cultured from the mouse forebrain; in addition, this intronic region is connected to the Gli3 promoter by a long-range interaction mediated by RNApolII ([39] and figure 6*a*). A DNA segment, overlapping the SOX2 peak, drives expression of a lacZ transgene to the embryonic mouse forebrain [40] and (https://enhancer.lbl.gov) (figure 6*a*). We found that this Sox2-bound region, when connected to a minimal promoter and a luciferase reporter gene, and transfected in Neuro2a cells, is activated by increasing doses of a cotransfected Sox2-expressing vector in a dose-dependent way (figure 6*b*).

Cxcr4, downregulated in early (FoxG1-Cre) Sox2 mutants at E14.5 (figure 4*g*), is also functionally involved in the development of the hippocampus (Discussion). Of note, an enhancer active in the developing brain, located within an intron of the Dars gene, but connected to the Cxcr4 gene promoter by a long-range interaction in brain-derived NSC chromatin, is bound by SOX2 in these cells [39] (figure 6*c*).

In conclusion, Sox2 early ablation leads to reduced expression, particularly in early (FoxG1-Cre) mutants, of several genes key to hippocampal development, some of which are directly bound and regulated by SOX2; some of these genes (Gli3, Wnt3a) are also known to functionally regulate each other (see Discussion). These genes may thus be considered as part of a Sox2-dependent gene regulatory network, controlling hippocampal development (figure 6*d*; see Discussion).

### 2.5. Emx1-Cre-mediated Sox2 ablation alters the excitatory input in CA3 and CA1 pyramidal neurons

We also investigated the consequences of Sox2 early loss on the physiological functioning of the postnatal hippocampus.

As illustrated earlier (figures 2 and 3), early Sox2 loss causes DG hypoplasia, most severe in FoxG1-cKO mutants, but clearly present also in Emx1-Cre cKO mice. Since FoxG1-cKO are perinatally lethal [9], we performed physiology studies on Emx1-cKO mutants. We addressed, in particular, the function of CA3 and CA1 pyramidal neurons, central to hippocampal circuitry and relatively spared, morphologically at least, in our mutants (in comparison to the severely hypoplastic DG).

**Figure 7.**
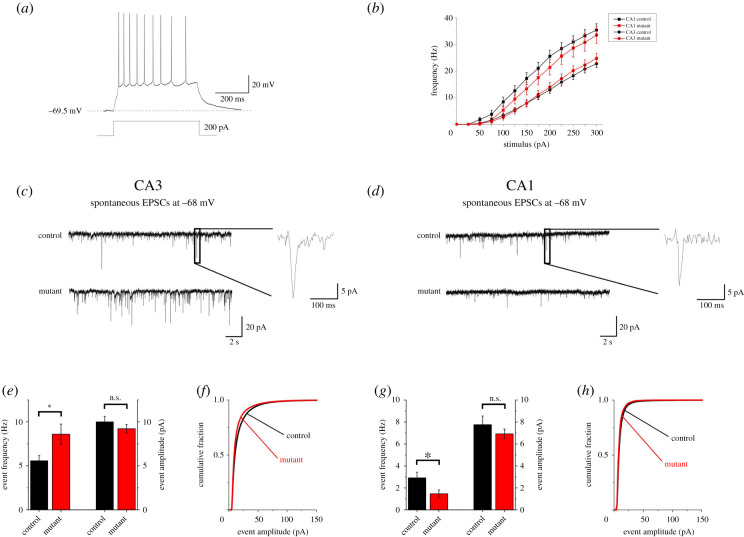
In Emx1-Cre cKO mice, excitatory transmission is altered in CA3 and CA1 hippocampal regions. Early Sox2 ablation leads to alterations in the excitatory input onto both CA3 and CA1 pyramidal neurons. (*a*) Typical firing response to a 200 pA stimulus of injected current in a CA3 pyramidal neuron. (*b*) Average stimulus/frequency relation for hippocampal pyramidal neurons recorded in CA3 *(circles)* and CA1 *(squares)*. No major differences were observed between control *(black)* and mutant animals *(red)*. (*c*,*d*) EPSCs traces, at −68 mV, recorded in simulated physiologic conditions onto pyramidal neurons in CA3 and CA1 region, respectively. *Insets.* Magnification of a representative EPSC event. (*e*) Average EPSCs frequencies and median amplitudes observed in CA3 pyramidal neurons recorded from 15 animals between p19 and p31. In mutant animals, Sox2 ablation induced a significant increase in EPSCs frequency compared to controls (8.60 ± 1.15 Hz, *n* = 20 and 5.59 ± 0.57 Hz, *n* = 28, respectively; *p* = 0.03941, with Mann–Whitney test), whereas no significant effect was produced on event amplitudes (9.21 ± 0.47 pA, *n* = 20 and 10.01 ± 0.61 pA, *n* = 28, respectively). (*f*) Amplitude distribution of the total amount of collected EPSCs showing no major differences between control and mutant mice. (*g*) In CA1, EPSCs frequency significantly decreased in mutant animals compared to controls (1.46 ± 0.35 Hz, *n* = 13 and 2.91 ± 0.52 Hz, *n* = 13, respectively; *p* = 0.02745, with Mann–Whitney test). No difference in the average median amplitude was observed (controls: 7.75 ± 0,69 pA, *n* = 20 and mutants: 6.92 ± 0.44 pA, *n* = 28). (*h*) The amplitude distribution of the total pool of events recorded from 13 animals between p19 and p31 showed no major alterations between control and mutant mice.

The DG receives its main extrinsic input from the entorhinal cortex and is the first hippocampal station of the classical trisynaptic pathway: entorhinal cortex → DG granule cells → CA3 pyramidal neurons → CA1 pyramidal neurons. The DG projects exclusively to CA3 through mossy fibres. In turn, CA3 projects to CA1 through Schaffer collaterals [41]. Hence, we investigated whether the hypoplastic DG in our Emx1-Cre mutants could alter signal transfer to CA3 and CA1. This hypothesis was tested by studying intrinsic excitability and excitatory transmission in CA3/CA1 pyramidal neurons. These were first identified by their typically large pyramidally shaped soma (approx. 20 µm diameter, in CA3), and then further distinguished by their action potential firing. We focused on regular-spiking pyramidal neurons, the widest population, characterized by slow firing with modest adaptation and excitability properties consistent with literature on CA1–CA3 neurons in mice (e.g. [24,42]). A typical example is shown in figure 7*a*. The excitability features of pyramidal neurons from control and mutant mice are shown in electronic supplementary material, table S1, while the stimulus/frequency relations are shown in figure 7*b*. Overall, little difference was observed in intrinsic excitability between mutant and control mice, in both CA1 and CA3.

In these neurons, we recorded the spontaneous excitatory post-synaptic currents (EPSCs) for 10 min after reaching the whole-cell configuration, at −68 mV. Spontaneous EPSCs reflect the overall excitatory input impinging on a given pyramidal neuron. Typical EPSC traces from CA3 pyramidal neurons are shown in figure 7*c*, for controls and mutants. Somewhat surprisingly, EPSC frequencies in CA3 displayed an approximately 30% increase in mutant animals compared to the controls (figure 7*e*). On the contrary, the average EPSC median amplitudes were not different between control and mutant mice (figure 7*e*). Moreover, the EPSC amplitudes obtained from all control and mutant cells are pooled in figure 7*f*. The amplitude distributions of the two genotypes were compared with KS test, which revealed no significant difference.

Next, we studied the excitatory input onto CA1, which is the last station of the hippocampal serial pathway of information transfer. Typical EPSC traces are shown in figure 7*d* for control and mutant. As expected [43], the overall EPSC frequency and amplitude tended to be smaller in CA1, compared to CA3. The average EPSC frequencies in CA1 are reported for control and mutant mice in figure 7*g*. Data reveal an approximately 50% reduction in mutant animals compared to the controls. Once again, little difference between genotypes was observed in the EPSC amplitudes (figure 7*g*,*h*).

In conclusion, CA3/CA1 pyramidal neuron firing or EPSC amplitudes were not altered in Emx1-Cre cKO mice, arguing against a direct effect of the mutation on the synaptic machinery or intrinsic excitability, which is consistent with the lack of expression of Sox2 in these neurons (data not shown). However, EPSC frequency increased in CA3 and was approximately halved in CA1 of mutant mice, suggesting that excitatory signal transfer along the canonical trisynaptic pathway was unbalanced as a consequence of the major impairment of DG development produced by Emx1-Cre-mediated Sox2 ablation.

Overall, our data indicate significant functional alterations of the hippocampal circuitry in Emx1-Cre Sox2 mutants, which might plausibly contribute to the epileptic and cognitive defects in human patients (see Discussion).

## 3. Discussion

In this work, we highlighted an early time window in hippocampal development, where the Sox2 transcription factor is necessary to initiate the embryogenesis of the hippocampus. In fact, following Sox2 deletion with FoxG1-Cre, active from E9.5 [9,14], hippocampal development is drastically defective, with a nearly complete absence of the DG; DG development is also defective, but present, following deletion with Emx1-Cre, active from E10.5 [22]; the later Sox2 deletion with Nestin-Cre [8] has very little effect on hippocampal embryogenesis (figures 2 and 3). We note that even the earliest Sox2 deletion (FoxG1-Cre), while essentially preventing the development of the DG, still allows the development of the CA1/3 hippocampal pyramidal neurons, although Sox2 is likely removed also from the CA1/3 precursors; this suggests the existence, already at early stages (E9.5–E10.5), of a distinct gene expression programme required for DG development, but not (or less so) for CA1/3 development.

As noted in Results, the deletion of Sox2 by FoxG1-Cre involves the simultaneous loss of a copy of FoxG1 [14]; thus, FoxG1 heterozygosity might, in principle, contribute to the observed defects, given that a homozygous FoxG1 null mutation leads to smaller cerebral hemispheres [25]. However, we fail to detect abnormalities (when compared to wild-type) in the FoxG1-Cre heterozygous Sox2 mutants (Sox2^+/flox^; FoxG1^+/Cre^), which are also heterozygous for FoxG1 loss, whereas we detect major defects in the FoxG1-Cre homozygous Sox2 mutants (Sox2^flox/flox^; FoxG1^+/Cre^). Thus, the absence of a single FoxG1 allele does not seem to influence, *per se*, the phenotype of our Sox2 mutants; the similarity of FoxG1-Cre heterozygous Sox2 mutants to wild-type littermates is in agreement with the absence of significant hippocampal abnormalities in E18.5 FoxG1+/− embryos reported by Shen *et al*. [44]. We thus attribute the major defects seen in FoxG1-Cre Sox2 mutants to the homozygous loss of Sox2. We cannot completely rule out, however, that the loss of a single FoxG1 allele somehow contributes, in the presence of a homozygous Sox2 loss, to an increased severity of the defect.

Our observations on FoxG1-Cre, Emx1-Cre and Nestin-Cre Sox2 mutants point to gene regulatory events, orchestrated by Sox2, that are required to initiate hippocampal development; at least some of these events are likely to be direct effects of SOX2 (figure 6). What is the nature of these events?

### 3.1. Gene regulatory events mediating early Sox2 function in hippocampal development

An important reduction in the early expression of key regulators of hippocampal development (Wnt3a; Gli3; Cxcr4; Tbr2; p73) is observed in early Sox2 mutants (FoxG1-Cre cKO), already at early stages of hippocampal embryogenesis (E12.5, E14.5), preceding the overt phenotypic manifestation of the defect (hippocampal morphogenesis begins at about E14.5) (figures 4 and 5). Of note, reduced expression in the mutant CH at E12.5 is seen for some genes (e.g. Wnt3a, Gli3), but not others (Wnt5a; figure 5), suggesting that the CH is present, but misfunctional in directing hippocampal formation. Importantly, a reduction in expression of these master genes is also observed in Emx1-Cre mutants, but to a lesser extent than in FoxG1-Cre mutants (figure 5). These observations suggest that the differential reduction in the expression of these key regulators accounts, at least in part, for the differences in the severity of the hippocampal embryogenesis defects between the three mutants.

What molecular mechanisms cause the differential expression of master hippocampal regulator genes between different mutants?

SOX2 is able to directly bind to at least some of these target genes (Gli3, Cxcr4, figure 6*a*,*c*) in neural cells chromatin, and to act as a transcriptional activator on some SOX2-bound enhancers (Gli3) within these loci (figure 6*b*), suggesting that it is directly involved in the transcriptional activation of at least some of these genes during hippocampal development. In addition, SOX2-bound distant enhancers within the Gli3 and Cxcr4 loci are connected to the gene promoter in a Sox2-dependent way, at least in NSC (figure 6*a*,*c*), indicating that SOX2 may contribute to their regulation also through this ‘architectural’ function [39,45].

At E12.5 and afterwards, Sox2 is ablated in both FoxG1-Cre and Emx1-Cre mutants, yet critical genes are much more downregulated in FoxG1-Cre mutants, in agreement with a requirement for Sox2 to properly initiate the expression of these genes at early stages. We speculate that SOX2 may act at early stages to initiate the organization of a 3D interaction network connecting gene promoters to enhancers (figure 6*a*,*c*), as a prerequisite for gene expression, in agreement with previous findings in NSC [39,45].

### 3.2. Altered regulation of a gene regulatory network of hippocampal master genes leads to defective cell development and cell–cell signalling in early Sox2 mutants, and eventually to defective hippocampal structure and function

The failure to properly activate early-acting hippocampal master genes may provide a molecular explanation for the failure to develop, in early Sox2 mutants, cell types essential in hippocampal development, or to prevent their proper behaviour, as observed in figures 2 and 3, leading to abnormal morphogenesis.

We note that Sox2 early mutation reduces the expression of several critical regulators of hippocampus development, known on the basis of the knock-out of the respective genes, but does not completely abolish it. Some of these downregulated genes are likely to represent direct targets of Sox2, such as Gli3 and Cxcr4 (figure 6). Accordingly, the effect of the downregulation of any individual gene may be expected to result in a less pronounced effect than the complete loss of the same gene, as observed in knock-out mice. In a complementary perspective, we propose that the phenotype observed in our early mutants results from the sum of the simultaneous deregulation of various different target genes, rather than from the loss of expression of just one or two critical targets; SOX2 being a transcription factor, we believe that this multi-target mode of action (see our model in figure 6*d*) more likely recapitulates the pathogenetic mechanism underlying the hippocampal defects in Sox2 mutants (and, we propose, in human patients with insufficient SOX2 dosage, such as that resulting from heterozygous SOX2 mutations, leading to hippocampal hypoplasia, see Introduction).

The Gli3 gene is a likely candidate for a Sox2 effector whose deregulation in Sox2 mutants may affect hippocampal development. Complete, germline Gli3 loss (homozygous null mutation) results in a failure of the medial wall of the telencephalon to invaginate to initiate hippocampal development, resulting in the absence of hippocampus formation [46,47]. We propose that partial inactivation of Gli3 expression (figure 5), in the FoxG1-Cre mutant, may contribute to the phenotype that we observe in our mutants, less severe than that of the Gli3 null mutants. In Gli3 mutant mice, the expression of Wnt signalling molecules, normally expressed in the CH, including Wnt3a and Wnt2b, is lost, and Wnt signalling is impaired at early stages of hippocampal development [38,48,49]. In the FoxG1-Cre Sox2 deletion mutant, both Wnt3a and Wnt2b are decreased (figure 5), suggesting that Gli3 decreased expression may be responsible for their downregulation, directly or indirectly. On the other hand, both our ChIPseq and ChIA-PET data [39] indicate that Sox2 is unlikely to act directly onto Wnt3a regulation.

Wnt signalling exerts its effects on target cells by inducing nuclear translocation of β-catenin, that acts as a transcriptional regulator associating with TCF transcription factors; mutation of TCF factors, e.g. Lef1, leads to failure of hippocampal development [46,50,51]. Of note, TCF binding regulates [52] the same intronic Gli3 enhancer, that we found to be bound and activated by Sox2 (figure 6*a*,*b*), suggesting that this element may integrate the effects of Wnt signalling and SOX2 activity in controlling Gli3 expression. Interestingly, Sox2/TCF binding sites were also described to act on other genes in the context of a transcriptional switch accompanying chromatin remodelling during neuronal differentiation [53].

We attempted to reactivate the Wnt pathway in the FoxG1-Cre cKO, by LiCl injection, to see if we could rescue any of the observed defects. We found some amelioration of the organization and number of CRC in the cortex (electronic supplementary material, figure S6B,C), although the overall hippocampus development remained defective (electronic supplementary material, figure S6A,C). We also tried to reactivate the Wnt pathway by a Wnt agonist (AZD1080 [54]); a partial rescue of Reelin retention in the CH, usually observed in mutants, was observed at E14.5 in the FoxG1-Cre cKO (electronic supplementary material, figure S6D). We hypothesized that earlier treatment might have had more pronounced effects; however, this resulted in high embryonic lethality, preventing us to observe the effects.

In conclusion, we propose that the loss of Wnt signalling from the CH represents one mechanism whereby Sox2 early loss causes defective hippocampal embryogenesis likely by regulating the production of CRC. Assessing the relative contribution of this mechanism will be postponed to future studies.

We detected, in our early mutants, reduced expression of Tbr2, Cxcr4, Cxcl12 and p73, marking specific cell types in hippocampal embryogenesis (figure 4). However, knock-out experiments previously demonstrated that these genes, in addition to marking specific cell types (figures 2 and 4), also play functional roles in hippocampal (as well as neocortical) development [19,35,55–57]. This suggests that their reduced expression in Sox2 mutants may also functionally contribute to the hippocampal defects.

Cxcr4, whose expression is downregulated at early stages in Sox2 early mutants (figure 4*g*), is essential in particular for the development of the DG [55,56]. Cxcr4 encodes a cell surface receptor, expressed in granule cell progenitors (GCP) of the developing hippocampus, that also express GFAP [56]. In hippocampal development, GCP, arising in the ventricular zone (DNE), migrate (DMS) to the subpial region, to form the granule cell layer (GCL) of the DG (figure 1*a*). The production and migration of GCP is regulated by various signalling molecules, including CXCL12 (the CXCR4 ligand), Reelin, Wnt and BMP proteins, secreted by regions surrounding the developing DG. In the absence of Cxcr4, the numbers of dividing cells in the migratory stream and the prospective DG are dramatically reduced [55]. It thus seems plausible that Cxcr4 deficiency importantly contributes to the impaired development of GFAP-positive GCP, and the consequent failure to develop a DG, seen in our early Sox2 mutants.

P73 encodes a transcription factor expressed in differentiating CRC (figure 4), the choroid plexus and the ependyma [58,59] and its knock-out in mice results in a phenotype very similar to the early loss of Sox2 in FoxG1-Cre cKO, with a lack of HF and almost absent DG [36]. P73 has a similar expression pattern in the fetal human brain suggesting a role in hippocampus development also in humans [36]. Interestingly, Reelin-expressing CRC are reduced in number and they may be retained in the CH instead of moving towards the pia in both Sox2 and P73 mutants. P73 has a very restricted expression pattern, but its knock-out has a broad effect on cortical patterning, suggesting it could be involved in the signalling activities of the CH [35].

### 3.3. Radial scaffold, Cajal–Retzius cells and lack of hippocampal fissure and dentate gyrus

One of the key outcomes of early ablation of Sox2 in the developing telencephalon, via FoxG1-Cre, is the lack of the hippocampal fissure followed by an extreme reduction of the DG. Radial glia scaffold disorganization due to knock-out of the transcription factor Nf1b leads to a lack of a specific hippocampal GFAP-positive glial population, lack of hippocampal fissure and DG without affecting cell proliferation, CRC differentiation or Wnt signalling [60]; this suggests that the loss and disorganization of GFAP-positive cells, seen in our mutants specifically in the developing hippocampus (figure 3), might constitute a cellular mechanism contributing to the defective DG development in early Sox2 mutants.

Interestingly, also the knock-out of P73 in CH-derived CRC cells leads to the lack of hippocampal fissure and DG, as previously mentioned [35]. CRC are known to regulate RG formation both in the cortex and in the developing hippocampus [61,62]; conversely, RG has been shown to be important for the correct positioning of CRC cells [63]. Our data suggest that Sox2 does not regulate proliferation in the medial telencephalon at E12.5 (electronic supplementary material, figure S5); it is possible that it regulates aspects of differentiation of RG and CRC.

### 3.4. Functional alteration of hippocampal circuitry in Sox2-ablated mice

In Emx1-Cre Sox2-deleted mice, we observed functional alterations in the excitatory transmission along the serial transmission pathway of the hippocampal formation, and particularly an inbalance in the excitatory input onto CA3 and CA1 pyramidal neurons (figure 7). Considering that (i) the main effects of Sox2 ablation are produced during hippocampal embryogenesis, (ii) Sox2 is not expressed in CA3/CA1 neurons, (iii) Sox2 deletion caused negligible alterations in pyramidal neuron excitability and excitatory synaptic efficacy, we attribute most of the observed functional effects to altered maturation of the connectivity pattern of hippocampal formation. Neural circuits in the hippocampal formation comprise both serial and parallel pathways. DG is regulated by cortical input from entorhinal layer III, and projects to CA3. However, entorhinal layer III also projects to CA3. Moreover, CA3 displays profuse recurrent reciprocal connections between pyramidal neurons [41]. Therefore, the higher EPSC frequency we observed in CA3 pyramidal neurons of Sox2-deleted mice could be caused by: (i) a denser innervation from entorhinal layer III, permitted by the lower entorhinal input to the hypoplastic DG; (ii) an increased recurrent collateral connectivity between CA3 cells, fostered by the absence of the physiological stimulus from DG; (iii) a decreased recurrent inhibition on CA3 pyramidal cells, as mossy fibres from DG also regulate GABAergic interneurons in CA3 [64]. We cannot presently distinguish between these mechanisms, which are not mutually exclusive. Nonetheless, the increased excitatory input we observed in CA3 pyramidal cells is consistent with the epileptic phenotype frequently associated with the brain malformations caused by Sox2 mutations [7]. Considering the peculiar propensity of CA3 region to develop seizure-like activity [65,66], we hypothesize that increased excitatory activity in CA3 of Sox2-deleted mice could facilitate seizure onset, perhaps through CA3 projection to septal areas [67,68].

By contrast, the excitatory input on CA1 pyramidal neurons was lower in Emx1-Cre cKO mice. This could be caused by increased local feedback inhibition by GABAergic neurons, because of overstimulation by the overactive CA3 fibres. Alternatively, in the absence of proper DG input, the partial disorganization of CA3 connectivity could favour recurrent collaterals at the expense of Schaffer collaterals. Regardless of the specific mechanism, our results demonstrate that Sox2 ablation at early developmental stages unbalances the normal CA3 to CA1 excitatory input, which could contribute to explain some of the cognitive alterations observed in Sox2 mutants. Although early Sox2 ablation leads to severe DG hypoplasia, many cognitive functions can be carried out even when hippocampal volume is strongly reduced [7,69]. It is, therefore, not surprising that the effects of Sox2 ablation on cognition of viable animals are subtle. Nonetheless, evidence is available in humans about a variety of cognitive alterations associated with Sox2 mutations [6,7]. In general, CA1 is the main output channel of the hippocampal formation, and is thought to compare the entorhinal cortex input (conveying the present state of the environment) with the CA3 input (conveying mnemonic representations of expected events based on external signals; [23]). Our results suggest that Sox2 malfunction may cause cognitive damage by altering such comparative function of CA1.

## 4. Conclusion and perspective

Overall, our work shows that Sox2 controls (directly, or indirectly) the activity of multiple, functionally interconnected genes, forming a gene regulatory programme active and required at very early stages of hippocampal development. Reduced activity of this programme leads to essentially absent (FoxG1-Cre mutants) or reduced (Emx1-Cre mutants) development of the hippocampus, in particular the DG. In the Emx1 mutants, which are viable, hippocampal physiology is importantly perturbed. These findings may provide novel perspectives for therapy approaches of genetic brain disease rooted in defective hippocampal development.

## 5. Material and methods

### 5.1. Mouse strains

Mutant embryos (Sox2^flox/flox^; Cre genotype) were obtained by crossing the Sox2Flox [8] line with the following lines: FoxG1-Cre [14], Emx1-Cre [15] and Nestin-Cre; Sox2βGeo [8,70,71].

For the experiments reported in electronic supplementary material, figure S1 (see below, *Lineage tracing of progeny of Sox2 expressing progenitors)*, the mouse line Sox2-CreERT2 [8] was crossed to a transgenic mouse line carrying a *loxP-EYFP* reporter of Cre activity (Rosa26R-EYFP) [72].

The day of vaginal plug was defined as embryonic day 0 (E0) and the day of birth as postnatal day 0 (P0).

Genotyping of adult mice or embryos was performed with the following primers:

Sox2 Flox Forward: 5′-AAGGTACTGGGAAGGGACATTT-3′

Sox2 Flox Reverse: 5′-AGGCTGAGTCGGGTCAATTA-3′

FoxG1-Cre Forward: 5′ AGTATTGTTTTGCCAAGTTCTAAT-3′

FoxG1-Cre Reverse: 5′-AGTATTGTTTTGCCAAGTTCTAAT-3′

Emx1-Cre IRES Forward: 5′-AGGAATGCAAGGTCTGTTGAAT-3′

Emx1-Cre IRES Reverse: 5′-TTTTTCAAAGGAAAACCACGTC-3′

Nestin-Cre Forward: 5′-CGCTTCCGCTGGGTCACTGTCG-3′

Nestin-Cre Reverse: 5′-TCGTTGCATCGACCGGTAATGCAGGC-3′

R26R-EYFP Forward: 5′-TTCCCGCACTAACCTAATGG-3′

R26R-EYFP Reverse: 5′-GAACTTCAGGGTCAGCTTGC-3′

Sox2-CreERT2 Forward: 5′-TGATCCTACCAGACCCTTCAGT-3′

Sox2-CreERT2 Reverse: 5′-TCTACACATTTTCCCTGGTTCC-3′

The FoxG1-Cre mouse line was maintained in 129 background as recommended in [14]. The other mouse lines were maintained in a mixed background enriched in C57BL/6 and DBA.

All procedures were in accordance with the European Communities Council Directive (2010/63/EU and 86/609/EEC), the National Institutes of Health guidelines and the Italian Law for Care and Use of Experimental Animals (DL26/14). They were approved by the Italian Ministry of Health and the Bioethical Committees of the University of Milan-Bicocca.

### 5.2. *In situ* hybridization

ISH was performed essentially as in [11]. Briefly, embryonic brains and P0 brains were dissected and fixed overnight (O/N) in paraformaldehyde 4% in PBS (phosphate-buffered saline; PFA 4%) at 4°C. The fixed tissue was cryoprotected in a series of sucrose solutions in PBS (15%, 30%) and then embedded in OCT (Killik, Bio-Optica) and stored at −80°C. Brains were sectioned (20 µm) with a cryostat, placed on a slide (Super Frost Plus 09-OPLUS, Menzel) and stored at −80°C. Slides were then defrosted, fixed in formaldehyde 4% in PBS for 10 min, washed three times for 5 min in PBS, incubated for 10 min in acetylation solution (for 200 ml: 2.66 ml triethanolamine, 0.32 ml HCl 37%, 0.5 ml acetic anhydride 98%) with constant stirring and then washed three times for 5 min in PBS. Slides were placed in a humid chamber and covered with prehybridization solution (50% formamide, 5× SSC, 0.25 mg ml^−1^ tRNA, 5X Denhardt's, 0.5 µg ml^−1^ salmon sperm) for at least 2 h and then incubated in hybridization solution (fresh prehybridization solution containing the digoxygenin (DIG)-labelled RNA probe of interest) O/N at 65°C. Slides were washed 5 min in 5× SSC, incubated two times in 0.2× SSC for 30 min at 65°C, washed 5 min in 0.2× SSC at room temperature (RT) and then 5 min in maleic acid buffer (MAB, 100 mM maleic acid, 150 mM NaCl pH 7.5). The slides were incubated in blocking solution (10% sheep serum, 2% blocking reagent (Roche), 0.3% Tween-20 in MAB) for at least 1 h at RT, then covered with fresh blocking solution containing anti-DIG antibody Roche 1 : 2000 and finally placed O/N at 4°C. Slides were washed in MAB three times for 5 min, in NTMT solution (100 mM NaCl, 100 mM Tris–HCl pH 9.5, 50 mM MgCl_2_, 0.1% Tween-20) two times for 10 min and then placed in a humid chamber, covered with BM Purple (Roche), incubated at 37°C until desired staining was obtained (1–6 h), washed in water for 5 min, air-dried and mounted with Eukitt (Sigma).

The following DIG-labelled probes were used: *Sox2* [16], *Cadherin8* [31], *Tbr2* [73], *Reelin* (a gift from Luca Muzio, HSR Milan [74]), *NeuroD* [75], *CTIP2* [29], *Hes5* [76], *Cxcr4* [55], *Cxcl12* [55], *Wnt3A* [38], *Wnt2b* [38], *Wnt5a* [38], *Gli3* (a gift from Luca Muzio, HSR Milan [77])*, Lhx2* (a gift from Shubha Tole, Tata Institute Mumbai [78]), *P73* (a gift from Olivia Hanley, UZH). The *P73* and *Prox1* probes were transcribed directly from a PCR product, obtained from E12.5 cDNA, with the following primers: P73 Forward 5′-AGCAGCAGCTCCTACAGAGG-3′ and P73 Reverse 5′-TAATACGACTCACTATAGGGCCTTGGGAAGTGAAGCACTC-3′ (which includes the T7 promoter underlined); Prox1 Forward 5′-TATATATTTGTGTGGGGGAGGC-3′ and Prox1 Reverse 5'-TAATACGACTCACTATAGGGGCAACTAGTGACAAAGCACAGG-3′ (which includes the T7 promoter underlined). Prox1 PCR primers sequences were taken from Allen Brain Atlas.

### 5.3. Quantification of pixel intensity of Gli3 ISH-stained sections

To quantify the *Gli3* ISH staining signal on coronal sections at E12.5 (figure 5*h*; electronic supplementary material, figure S3A), we converted all images to 8-bit greyscale with Fiji as in [79]. We drew a square (region of interest, ROI) and positioned it in the CH, the dentate neuroepithelium (DNE) or a region without ISH staining signal and measured the pixel intensity of each ROI. The background intensity was subtracted from the CH ROI and DNE ROI. These measurements were repeated on three to four sections from each brain sample. Data are represented as mean ± s.d. and were statistically analysed using unpaired Student's *t*-test, ***p < 0.005.

### 5.4. Immunohistochemistry

Immunohistochemistry was performed essentially as in [10]. Brains were dissected, fixed, embedded and sectioned as for ISH, except for fixation in PFA4% that was often 3–4 h at 4°C. Sections were washed in PBS 5 min, unmasked in citrate buffer (Na Citrate 0.01 M, Citric acid 0.01 M pH6) by boiling in a microwave 3 min and then washed in PBS 10 min at RT. Sections were blocked with blocking solution (FBS 10%, Triton 0.3%, PBS1X) for 1 h at RT, then incubated O/N in blocking solution with primary antibodies: anti-mSOX2 (R&D Systems MA2018, 1 : 50), anti-P73 (Neomarkers, 1:150), anti-Reelin (Millipore MAB5364, 1:500), anti-Tuj1 (Covance, 1:400), anti-GFP (Invitrogen A10262, 1 : 500, used to detect EYFP expressing cells), anti-GFAP (Dako, 1:500). Slides were then washed in PBS two times, 10 min each, and incubated in blocking solution containing the secondary fluorescent antibody (1 : 1000, Alexa Fluor Invitrogen) for 1 h 30 min at RT. Slides were then washed in PBS twice, 10 min each, and then mounted with Fluoromount (F4680, Sigma) with 4′,6-diamidino-2-phenylindole (DAPI) and imaged with a confocal microscope (Nikon A1R) and with a Zeiss Axioplan 2 fluorescent microscope for anti-GFAP immunostainings.

### 5.5. Lineage tracing of progeny of Sox2-expressing progenitors

R26R-EYFP females were crossed with Sox2-CreERT2 males. E9.5 pregnant females were injected intraperitoneally with tamoxifen (20 mg ml^−1^ in ethanol/corn oil 1 : 10, 0.1 mg g^−1^ of body weight) that induces Cre recombinase activity in the Sox2 telencephalic expression domain [8] and, therefore, turns on EYFP in this expression domain. Embryos were collected at E15.5, fixed in 4% PFA O/N, embedded in OCT and sectioned at the cryostat (20 µm sections) as for ISH (see above).

### 5.6. EdU tracing

Ethynyldeoxyuridine (EdU, Molecular Probes) was injected in E12.5 pregnant females at 50 µg g^−1^ body weight. Embryos were collected 30 min after injection, fixed O/N in PFA 4% and embedded for cryostat sectioning as above. Edu incorporation was detected on sections (20 µm) with the Click-iT EdU Kit Alexa Fluor 594 (C10354, Thermo Fisher) following the manufacturer's instructions. Briefly, slides were washed twice in PBS 2 min each and incubated for 20 min at RT in Triton 0.5% in PBS. Slides were then washed in Triton 0.1% in PBS three times, 3 min each. Sections were incubated 30 min in the dark with EdU Click reaction according to the manufacturer's instructions. Slides were then washed in PBS three times 5 min, stained with DAPI, mounted with Fluoromount (F4680, Sigma) and imaged with a confocal microscope (Nikon A1R). The number of EdU-positive cells in the CH and dentate neuroephitelium was counted on at least three consecutive coronal sections for each brain. Data are represented as mean ± s.d.

### 5.7. Brain slices

For patch-clamp experiments, coronal sections (300 µm thick) containing the hippocampal region (−1.22 to −2.70 mm from bregma) were prepared from mice of both sexes (6 male and 10 female) aged P19–P31, by applying standard procedures [80].

### 5.8. Patch-clamp recording and data analysis

Cells were examined with an Eclipse E600FN direct microscope, equipped with water immersion DIC objective (Nikon Instruments, Milano, Italy), and digital CCD C8484-05G01 IR camera with HCImage Live acquisition software (Hamamatsu Photonics Italia, Arese, Italy). Stimulation and recording were carried out in whole-cell mode, by using a Multiclamp 700A amplifier (Molecular Devices, Sunnyvale, CA, USA), at 33–34°C. Borosilicate capillaries (OD 1.5 mm; Corning Inc., NY, USA) were pulled (2–3 MΩ) with a Flaming/Brown P-97 micropipette puller (Sutter Instruments, Novato, CA, USA). Series resistance after patch rupture was usually around 10–15 MΩ and was compensated up to at least 70%. Cell capacitance was also compensated. Synaptic currents and action potentials were low-pass filtered a 2 kHz and digitized at 5 kHz with Digidata 1322A/pClamp 9.2 (Molecular Devices). During recording, slices were perfused (approx. 2 ml min^−1^) with artificial cerebrospinal fluid, containing (mM): 135 NaCl, 21 NaHCO_3_, 0.6 CaCl_2_, 3 KCl, 1.25 NaH_2_PO_4_, 1.8 MgSO_4_, 10 d-glucose, aerated with 95% O_2_ and 5% CO_2_ (pH 7.4). Pipette contained (mM): 140 K-gluconate, 5 KCl, 1 MgCl_2_, 0.5 BAPTA, 1 MgATP, 0.3 NaGTP, 10 HEPES (pH 7.26). Resting membrane potential (*V*_rest_) was determined in open circuit mode (*I* = 0), immediately after reaching the whole-cell configuration. No correction was applied for liquid junction potentials. Series resistance was monitored throughout the experiment by applying small stimuli around *V*_rest_. Cells were discarded when *R*s was higher than 15 MΩ.

Action potentials and EPSCs were analysed with Clampfit 9.2 (Molecular Devices), MiniAnalysis and OriginPro 9.1 (OriginLab Corporation, Northampton, MA, USA), as previously reported [80,81].

### 5.9. AZD1080 and LiCl treatment

AZD1080 (Axon Medchem, Axon Catalogue ID: 2171) diluted in ascorbic acid 0.5%/EDTA 0.01% [54], was administered to pregnant females once a day by oral gavage from E9.5 to E12.5. We administered 5 µl AZD1080/g of body weight (AZD1080 0.375 µg µl^−1^ at E9.5 and E10.5, AZD1080 0.75 µg µl^−1^ at E11.5 and E12.5). Embryos were then collected at E14.5. Ascorbic acid 0.5%/EDTA 0.01% was administered as a control.

LiCl, or NaCl as a control, were injected intraperitoneally in pregnant female from E9.5 to E14.5 or from E10.5 to E12.5 once a day at the same time. No difference was observed between the two injection time windows. Ten microlitres per gram of body weight of 600 mM LiCl or 600 mM NaCl were injected. Embryos were collected at E18.5 and processed for ISH. The injection of AZD1080 or LiCl in pregnant females at E8.5 led to abortions.

The number of *Reelin*-positive cells at the hippocampal fissure and in the cortex was counted using Photoshop CC 2015 on five consecutive coronal sections of each brain. Data are represented as mean ± s.d. and were statistically analysed using unpaired Student's *t*-test, ***p < 0.005.

### 5.10. Luciferase constructs

The DNA region in the Gli3 second intron overlapping the SOX2 peak, and corresponding to the VISTA enhancer (coordinates under the embryo in figure 6*a*) was PCR-amplified from the vector where it had been cloned upstream to the lacZ reporter (a gift from T. Theil; [52]), and cloned upstream to the tk promoter in the Tk-luc vector [18], into the KpnI and NheI restriction sites.

### 5.11. Genomic SOX2 ChIPseq and ChIA-PET datasets used in figure 6

The ChIPseq and ChIA-PET genomic data [39] have been deposited in NCBI's Gene Expression Omnibus (GEO) with accession number GEO: GSE90561, and can be visualized through the WashU Epigenome Browser at: http://epigenomegateway.wustl.edu/legacy/?genome=mm9&datahub=https://wangftp.wustl.edu/dli/7131149234337a58201ae3da174ecc51/hub&coordinate=chr8:87120161-87587163.

### 5.12. Transfection experiments

The transfection experiments were performed essentially as previously described [11,82]. In particular, Neuro2a cells were plated in Minimal Essential Medium Eagle (MEM; SIGMA), supplemented with 10% fetal bovine serum, l-glutamine, penicillin and streptomycin. For transfection, cells were plated in 12-well plates at 1.5 × 10^5^ cells well^−1^, and transfected on the following day using Lipofectamine 2000 (Invitrogen). Briefly, medium in each well was replaced with 1 ml of MEM medium (with no addition) mixed with 2 µl of Lipofectamine 2000, and DNA. After 4 h from transfection, the medium was replaced with complete medium. A fixed amount of 300 ng of luciferase reporter plasmid was used for each well, with increasing amounts of Sox2-expressing vector [8,18], or the corresponding control ‘empty’ vector (not containing the transcription factor's cDNA), in the following luciferase vector : expressing vector molar ratios (indicated in figure 6): +, 1:0.050; ++, 1:0.075; +++, 1:0.125; ++++, 1:0.25; +++++, 1:0.5. The pBluescript vector was added to transfection DNA to equalize the total amount of transfected DNA to a total of 800 ng for each reaction. After 24 h, total cellular extracts were prepared, and luciferase activity was measured with a Promega Luciferase Assay System, according to the manufacturer's instructions.

## Data accessibility

The genomic data related to figure 6 (see [35]) have been deposited in NCBI's Gene Expression Omnibus (GEO) and are available with accession number GEO: GSE90561. Data can be visualized through the WashU browser: http://epigenomegateway.wustl.edu/legacy/?genome=mm9&datahub=https://wangftp.wustl.edu/_dli/7131149234337a58201ae3da174ecc51/hub&coordinate=chr8:87120161-87587163.

## References

[1] Kandel ER, Schwartz JH, Jessell TM. 2000 Principles of neural science, 4th edn. New York, NY: McGraw-Hill, Health Professions Division.

[2] Berg DA et al. 2019 A common embryonic origin of stem cells drives developmental and adult neurogenesis. Cell **177**, 654-668 e15. (10.1016/j.cell.2019.02.010) 30929900 PMC6496946

[3] Zhong S et al. 2020 Decoding the development of the human hippocampus. Nature **577**, 531-536. (10.1038/s41586-019-1917-5) 31942070

[4] Fantes J et al. 2003 Mutations in SOX2 cause anophthalmia. Nat. Genet. **33**, 461-463. (10.1038/ng1120) 12612584

[5] Kondoh H L-BReb. 2016 Sox2, biology and role in development and disease. London, UK: Academic Press.

[6] Ragge NK et al. 2005 SOX2 anophthalmia syndrome. Am. J. Med. Genet. A **135**, 1-7. discussion 8. (10.1002/ajmg.a.30642) 15812812

[7] Sisodiya SM et al. 2006 Role of SOX2 mutations in human hippocampal malformations and epilepsy. Epilepsia **47**, 534-542. (10.1111/j.1528-1167.2006.00464.x) 16529618

[8] Favaro R et al. 2009 Hippocampal development and neural stem cell maintenance require Sox2-dependent regulation of Shh. Nat. Neurosci. **12**, 1248-1256. (10.1038/nn.2397) 19734891

[9] Ferri A et al. 2013 Sox2 is required for embryonic development of the ventral telencephalon through the activation of the ventral determinants Nkx2.1 and Shh. Development **140**, 1250-1261. (10.1242/dev.073411) 23444355

[10] Cerrato V et al. 2018 Sox2 conditional mutation in mouse causes ataxic symptoms, cerebellar vermis hypoplasia, and postnatal defects of Bergmann glia. Glia **66**, 1929-1946. (10.1002/glia.23448) 29732603

[11] Mercurio S et al. 2019 Sox2 acts in thalamic neurons to control the development of retina–thalamus–cortex connectivity. iScience **15**, 257-273. (10.1016/j.isci.2019.04.030) 31082736 PMC6517317

[12] Mercurio S, Serra L, Nicolis SK. 2019 More than just stem cells: functional roles of the transcription factor Sox2 in differentiated glia and neurons. Int. J. Mol. Sci. **20**, 4540. (10.3390/ijms20184540) 31540269 PMC6769708

[13] Rogers N, Cheah PS, Szarek E, Banerjee K, Schwartz J, Thomas P. 2013 Expression of the murine transcription factor SOX3 during embryonic and adult neurogenesis. Gene Expr. Patterns **13**, 240-248. (10.1016/j.gep.2013.04.004) 23665444

[14] Hébert JM, McConnell SK. 2000 Targeting of cre to the Foxg1 (BF-1) locus mediates loxP recombination in the telencephalon and other developing head structures. Dev. Biol. **222**, 296-306. (10.1006/dbio.2000.9732) 10837119

[15] Gorski JA, Talley T, Qiu M, Puelles L, Rubenstein JL, Jones KR. 2002 Cortical excitatory neurons and glia, but not GABAergic neurons, are produced in the Emx1-expressing lineage. J. Neurosci. **22**, 6309-6314. (10.1523/JNEUROSCI.22-15-06309.2002) 12151506 PMC6758181

[16] Avilion AA, Nicolis SK, Pevny LH, Perez L, Vivian N, Lovell-Badge R. 2003 Multipotent cell lineages in early mouse development depend on SOX2 function. Genes Dev. **17**, 126-140. (10.1101/gad.224503) 12514105 PMC195970

[17] Ferri AL et al. 2004 Sox2 deficiency causes neurodegeneration and impaired neurogenesis in the adult mouse brain. Development **131**, 3805-3819. (10.1242/dev.01204) 15240551

[18] Mariani J et al. 2012 Emx2 is a dose-dependent negative regulator of Sox2 telencephalic enhancers. Nucleic Acids Res. **40**, 6461-6476. (10.1093/nar/gks295) 22495934 PMC3413107

[19] Hodge RD, Garcia III AJ, Elsen GE, Nelson BR, Mussar KE, Reiner SL, Ramirez J-M, Hevner RF. 2013 Tbr2 expression in Cajal–Retzius cells and intermediate neuronal progenitors is required for morphogenesis of the dentate gyrus. J. Neurosci. **33**, 4165-4180. (10.1523/JNEUROSCI.4185-12.2013) 23447624 PMC3623668

[20] Grove EA. 2008 Neuroscience. Organizing the source of memory. Science **319**, 288-289. (10.1126/science.1153743) 18202278

[21] Mangale VS et al. 2008 Lhx2 selector activity specifies cortical identity and suppresses hippocampal organizer fate. Science **319**, 304-309. (10.1126/science.1151695) 18202285 PMC2494603

[22] Shetty AS et al. 2013 Lhx2 regulates a cortex-specific mechanism for barrel formation. Proc. Natl Acad. Sci. USA **110**, E4913-E4921. (10.1073/pnas.1311158110) 24262147 PMC3864327

[23] Knierim JJ, Neunuebel JP. 2016 Tracking the flow of hippocampal computation: pattern separation, pattern completion, and attractor dynamics. Neurobiol. Learn. Mem. **129**, 38-49. (10.1016/j.nlm.2015.10.008) 26514299 PMC4792674

[24] Venkatesan K, Liu Y, Goldfarb M. 2014 Fast-onset long-term open-state block of sodium channels by A-type FHFs mediates classical spike accommodation in hippocampal pyramidal neurons. J. Neurosci. **34**, 16 126-16 139. (10.1523/JNEUROSCI.1271-14.2014) PMC4244476 25429153

[25] Xuan S, Baptista CA, Balas G, Tao W, Soares VC, Lai E. 1995 Winged helix transcription factor BF-1 is essential for the development of the cerebral hemispheres. Neuron **14**, 1141-1152. (10.1016/0896-6273(95)90262-7) 7605629

[26] Li G, Kataoka H, Coughlin SR, Pleasure SJ. 2009 Identification of a transient subpial neurogenic zone in the developing dentate gyrus and its regulation by Cxcl12 and reelin signaling. Development **136**, 327-335. (10.1242/dev.025742) 19103804 PMC2685973

[27] Basak O, Taylor V. 2007 Identification of self-replicating multipotent progenitors in the embryonic nervous system by high Notch activity and Hes5 expression. Eur. J. Neurosci. **25**, 1006-1022. (10.1111/j.1460-9568.2007.05370.x) 17331197

[28] Iwano T, Masuda A, Kiyonari H, Enomoto H, Matsuzaki F. 2012 Prox1 postmitotically defines dentate gyrus cells by specifying granule cell identity over CA3 pyramidal cell fate in the hippocampus. Development **139**, 3051-3062. (10.1242/dev.080002) 22791897

[29] Leid M, Ishmael JE, Avram D, Shepherd D, Fraulob V, Dolle P. 2004 CTIP1 and CTIP2 are differentially expressed during mouse embryogenesis. Gene Expr. Patterns **4**, 733-739. (10.1016/j.modgep.2004.03.009) 15465497 PMC2819357

[30] Simon R et al. 2012 A dual function of Bcl11b/Ctip2 in hippocampal neurogenesis. EMBO J **31**, 2922-2936. (10.1038/emboj.2012.142) 22588081 PMC3395096

[31] Korematsu K, Redies C. 1997 Expression of cadherin-8 mRNA in the developing mouse central nervous system. J. Comp. Neurol. **387**, 291-306. (10.1002/(SICI)1096-9861(19971020)387:2<291::AID-CNE10>3.0.CO;2-Y) 9336230

[32] D'Arcangelo G, Miao GG, Chen SC, Soares HD, Morgan JI, Curran T. 1995 A protein related to extracellular matrix proteins deleted in the mouse mutant reeler. Nature **374**, 719-723. (10.1038/374719a0) 7715726

[33] Berger O, Li G, Han SM, Paredes M, Pleasure SJ. 2007 Expression of SDF-1 and CXCR4 during reorganization of the postnatal dentate gyrus. Dev. Neurosci. **29**, 48-58. (10.1159/000096210) 17148948

[34] Borrell V, Marin O. 2006 Meninges control tangential migration of hem-derived Cajal–Retzius cells via CXCL12/CXCR4 signaling. Nat. Neurosci. **9**, 1284-1293. (10.1038/nn1764) 16964252

[35] Meyer G, Cabrera Socorro A, Perez Garcia CG, Martinez Millan L, Walker N, Caput D. 2004 Developmental roles of p73 in Cajal–Retzius cells and cortical patterning. J. Neurosci. **24**, 9878-9887. (10.1523/JNEUROSCI.3060-04.2004) 15525772 PMC6730229

[36] Meyer G, Gonzalez-Arnay E, Moll U, Nemajerova A, Tissir F, Gonzalez-Gomez M. 2019 Cajal–Retzius neurons are required for the development of the human hippocampal fissure. J. Anat. **235**, 569-589. (10.1111/joa.12947) 30861578 PMC6704247

[37] Lee SM, Tole S, Grove E, McMahon AP. 2000 A local Wnt-3a signal is required for development of the mammalian hippocampus. Development **127**, 457-467. 10631167 10.1242/dev.127.3.457

[38] Grove EA, Tole S, Limon J, Yip L, Ragsdale CW. 1998 The hem of the embryonic cerebral cortex is defined by the expression of multiple Wnt genes and is compromised in Gli3-deficient mice. Development **125**, 2315-2325. 9584130 10.1242/dev.125.12.2315

[39] Bertolini JA et al. 2019 Mapping the global chromatin connectivity network for Sox2 function in neural stem cell maintenance. Cell Stem Cell. **24**, 462-476e6. (10.1016/j.stem.2019.02.004) 30849367 PMC6506828

[40] Visel A et al. 2009 ChIP-seq accurately predicts tissue-specific activity of enhancers. Nature **457**, 854-858. (10.1038/nature07730) 19212405 PMC2745234

[41] Witter MP, Amaral DG. 2004. Hippocampal formation. In The rat nervous system (ed. E Paxinos), pp. 635-704, 3rd edn. Amsterdam, The Netherlands: Elsevier.

[42] Hunt DL, Linaro D, Si B, Romani S, Spruston N. 2018 A novel pyramidal cell type promotes sharp-wave synchronization in the hippocampus. Nat. Neurosci. **21**, 985-995. (10.1038/s41593-018-0172-7) 29915194

[43] Traub RD, Jefferys JGR, Whittington MA. 1999 Fast oscillations in cortical circuits. Cambridge, MA: MIT Press.

[44] Shen L, Nam HS, Song P, Moore H, Anderson SA. 2006 FoxG1 haploinsufficiency results in impaired neurogenesis in the postnatal hippocampus and contextual memory deficits. Hippocampus **16**, 875-890. (10.1002/hipo.20218) 16941454

[45] Wei CL, Nicolis SK, Zhu Y, Pagin M. 2019 Sox2-dependent 3D chromatin interactomes in transcription, neural stem cell proliferation and neurodevelopmental diseases. J. Exp. Neurosci. **13**, 1179069519868224. (10.1177/1179069519868224) 31431802 PMC6686325

[46] Li G, Pleasure SJ. 2014 The development of hippocampal cellular assemblies. Wiley Interdiscip. Rev. Dev. Biol. **3**, 165-177. (10.1002/wdev.127) 24719288

[47] Theil T, Alvarez-Bolado G, Walter A, Ruther U. 1999 Gli3 is required for Emx gene expression during dorsal telencephalon development. Development **126**, 3561-3571. 10409502 10.1242/dev.126.16.3561

[48] Fotaki V, Price DJ, Mason JO. 2011 Wnt/beta-catenin signaling is disrupted in the extra-toes (Gli3(Xt/Xt)) mutant from early stages of forebrain development, concomitant with anterior neural plate patterning defects. J. Comp. Neurol. **519**, 1640-1657. (10.1002/cne.22592) 21452227

[49] Theil T, Aydin S, Koch S, Grotewold L, Ruther U. 2002 Wnt and Bmp signalling cooperatively regulate graded Emx2 expression in the dorsal telencephalon. Development **129**, 3045-3054. 12070081 10.1242/dev.129.13.3045

[50] Galceran J, Miyashita-Lin EM, Devaney E, Rubenstein JL, Grosschedl R. 2000 Hippocampus development and generation of dentate gyrus granule cells is regulated by LEF1. Development **127**, 469-482. 10631168 10.1242/dev.127.3.469

[51] Roelink H. 2000 Hippocampus formation: an intriguing collaboration. Curr. Biol. **10**, R279-R281. (10.1016/S0960-9822(00)00407-3) 10753739

[52] Hasenpusch-Theil K, Magnani D, Amaniti EM, Han L, Armstrong D, Theil T. 2012 Transcriptional analysis of Gli3 mutants identifies Wnt target genes in the developing hippocampus. Cereb. Cortex **22**, 2878-2893. (10.1093/cercor/bhr365) 22235033 PMC3491769

[53] Muotri AR, Marchetto MC, Coufal NG, Oefner R, Yeo G, Nakashima K, Gage FH. 2010 L1 retrotransposition in neurons is modulated by MeCP2. Nature **468**, 443-446. (10.1038/nature09544) 21085180 PMC3059197

[54] Georgievska B et al. 2013 AZD1080, a novel GSK3 inhibitor, rescues synaptic plasticity deficits in rodent brain and exhibits peripheral target engagement in humans. J. Neurochem. **125**, 446-456. (10.1111/jnc.12203) 23410232

[55] Lu M, Grove EA, Miller RJ. 2002 Abnormal development of the hippocampal dentate gyrus in mice lacking the CXCR4 chemokine receptor. Proc. Natl Acad. Sci. USA **99**, 7090-7095. (10.1073/pnas.092013799) 11983855 PMC124533

[56] Mimura-Yamamoto Y, Shinohara H, Kashiwagi T, Sato T, Shioda S, Seki T. 2017 Dynamics and function of CXCR4 in formation of the granule cell layer during hippocampal development. Sci. Rep. **7**, 5647. (10.1038/s41598-017-05738-7) 28717168 PMC5514042

[57] Bagri A, Gurney T, He X, Zou YR, Littman DR, Tessier-Lavigne M, Pleasure SJ. 2002 The chemokine SDF1 regulates migration of dentate granule cells. Development **129**, 4249-4260. 12183377 10.1242/dev.129.18.4249

[58] Meyer G, Schaaps JP, Moreau L, Goffinet AM. 2000 Embryonic and early fetal development of the human neocortex. J. Neurosci. **20**, 1858-1868. (10.1523/JNEUROSCI.20-05-01858.2000) 10684887 PMC6772901

[59] Yang A et al. 2000 p73-deficient mice have neurological, pheromonal and inflammatory defects but lack spontaneous tumours. Nature **404**, 99-103. (10.1038/35003607) 10716451

[60] Barry G et al. 2008 Specific glial populations regulate hippocampal morphogenesis. J. Neurosci. **28**, 12 328-12 340. (10.1523/JNEUROSCI.4000-08.2008) PMC6671696 19020026

[61] Forster E, Tielsch A, Saum B, Weiss KH, Johanssen C, Graus-Porta D, Muller U, Frotscher M. 2002 Reelin, disabled 1, and beta 1 integrins are required for the formation of the radial glial scaffold in the hippocampus. Proc. Natl Acad. Sci. USA **99**, 13 178-13 183. (10.1073/pnas.202035899) 12244214 PMC130606

[62] Frotscher M, Haas CA, Forster E. 2003 Reelin controls granule cell migration in the dentate gyrus by acting on the radial glial scaffold. Cereb. Cortex. **13**, 634-640. (10.1093/cercor/13.6.634) 12764039

[63] Kwon HJ, Ma S, Huang Z. 2011 Radial glia regulate Cajal–Retzius cell positioning in the early embryonic cerebral cortex. Dev. Biol. **351**, 25-34. (10.1016/j.ydbio.2010.12.026) 21185282

[64] Acsady L, Kamondi A, Sik A, Freund T, Buzsaki G. 1998 GABAergic cells are the major postsynaptic targets of mossy fibers in the rat hippocampus. J. Neurosci. **18**, 3386-3403. (10.1523/JNEUROSCI.18-09-03386.1998) 9547246 PMC6792657

[65] de la Prida LM, Huberfeld G, Cohen I, Miles R. 2006 Threshold behavior in the initiation of hippocampal population bursts. Neuron **49**, 131-142. (10.1016/j.neuron.2005.10.034) 16387645

[66] Miles R, Wong RK. 1983 Single neurones can initiate synchronized population discharge in the hippocampus. Nature **306**, 371-373. (10.1038/306371a0) 6316152

[67] Colom LV. 2006 Septal networks: relevance to theta rhythm, epilepsy and Alzheimer's disease. J. Neurochem. **96**, 609-623. (10.1111/j.1471-4159.2005.03630.x) 16405497

[68] Swanson LW, Cowan WM. 1977 An autoradiographic study of the organization of the efferent connections of the hippocampal formation in the rat. J. Comp. Neurol. **172**, 49-84. (10.1002/cne.901720104) 65364

[69] Moser MB, Moser EI. 1998 Functional differentiation in the hippocampus. Hippocampus **8**, 608-619. (10.1002/(SICI)1098-1063(1998)8:6<608::AID-HIPO3>3.0.CO;2-7) 9882018

[70] Medina DL, Sciarretta C, Calella AM, Von Bohlen Und Halbach O, Unsicker K, Minichiello L. 2004 TrkB regulates neocortex formation through the Shc/PLCgamma-mediated control of neuronal migration. EMBO J. **23**, 3803-3814. (10.1038/sj.emboj.7600399) 15372074 PMC522798

[71] Tronche F, Kellendonk C, Kretz O, Gass P, Anlag K, Orban PC, Bock R, Klein R, Schütz G. 1999 Disruption of the glucocorticoid receptor gene in the nervous system results in reduced anxiety. Nat. Genet. **23**, 99-103. (10.1038/12703) 10471508

[72] Srinivas S, Watanabe T, Lin CS, William CM, Tanabe Y, Jessell TM, Costantini F. 2001 Cre reporter strains produced by targeted insertion of EYFP and ECFP into the ROSA26 locus. BMC Dev. Biol. **1**, 4. (10.1186/1471-213X-1-4) 11299042 PMC31338

[73] Bulfone A, Martinez S, Marigo V, Campanella M, Basile A, Quaderi N, Gattuso C, Rubenstein JLR, Ballabio A. 1999 Expression pattern of the Tbr2 (Eomesodermin) gene during mouse and chick brain development. Mech. Dev. **84**, 133-138. (10.1016/S0925-4773(99)00053-2) 10473127

[74] Mallamaci A, Mercurio S, Muzio L, Cecchi C, Pardini CL, Gruss P, Boncinelli E. 2000 The lack of Emx2 causes impairment of Reelin signaling and defects of neuronal migration in the developing cerebral cortex. J. Neurosci. **20**, 1109-1118. (10.1523/JNEUROSCI.20-03-01109.2000) 10648716 PMC6774155

[75] Cau E, Gradwohl G, Fode C, Guillemot F. 1997 Mash1 activates a cascade of bHLH regulators in olfactory neuron progenitors. Development **124**, 1611-1621. 9108377 10.1242/dev.124.8.1611

[76] Machold RP, Kittell DJ, Fishell GJ. 2007 Antagonism between Notch and bone morphogenetic protein receptor signaling regulates neurogenesis in the cerebellar rhombic lip. Neural Dev. **2**, 5. (10.1186/1749-8104-2-5) 17319963 PMC1820780

[77] Schimmang T, Lemaistre M, Vortkamp A, Ruther U. 1992 Expression of the zinc finger gene Gli3 is affected in the morphogenetic mouse mutant extra-toes (Xt). Development **116**, 799-804. 1289066 10.1242/dev.116.3.799

[78] Bulchand S, Grove EA, Porter FD, Tole S. 2001 LIM-homeodomain gene Lhx2 regulates the formation of the cortical hem. Mech. Dev. **100**, 165-175. (10.1016/S0925-4773(00)00515-3) 11165475

[79] Dobrzycki T, Krecsmarik M, Monteiro R. 2020 Genotyping and quantification of *in situ* hybridization staining in zebrafish. J. Vis. Exp. **155**, e59956. (10.3791/59956) 32065138

[80] Aracri P, Meneghini S, Coatti A, Amadeo A, Becchetti A. 2017 α4β2(*) nicotinic receptors stimulate GABA release onto fast-spiking cells in layer V of mouse prefrontal (Fr2) cortex. Neuroscience **340**, 48-61. (10.1016/j.neuroscience.2016.10.045) 27793780 PMC5231322

[81] Aracri P, Amadeo A, Pasini ME, Fascio U, Becchetti A. 2013 Regulation of glutamate release by heteromeric nicotinic receptors in layer V of the secondary motor region (Fr2) in the dorsomedial shoulder of prefrontal cortex in mouse. Synapse **67**, 338-357. (10.1002/syn.21655) 23424068

[82] Panaliappan TK et al. 2018 Sox2 is required for olfactory pit formation and olfactory neurogenesis through BMP restriction and Hes5 upregulation. Development **145**, 1-13. (10.1242/dev.153791) PMC5825848 29352015

